# Using 10-K text to gauge COVID-related corporate disclosure

**DOI:** 10.1371/journal.pone.0283138

**Published:** 2023-03-22

**Authors:** Shantanu Dutta, Ashok Kumar, Pushpesh Pant, Caolan Walsh, Moumita Dutta

**Affiliations:** 1 Telfer School of Management, University of Ottawa, Ottawa, Canada; 2 Operations Department, IMT Hyderabad, Shamshabad, Telangana, India; 3 Department of Surgery, Faculty of Medicine, University of Ottawa, Ottawa, Canada; 4 Faculty of Science, University of Ottawa, Ottawa, Canada; Yunnan Technology and Business University, CHINA

## Abstract

During the pandemic era, COVID-related disclosure has become quite critical for shareholders and other market participants to understand the uncertainties and challenges associated with a firm’s operation. However, there is no well-grounded and systematic measure to gauge the intensity of COVID-related disclosure and its plausible impact. Therefore, this study develops and validates various COVID-related disclosure measures. More specifically, using a sample of publicly listed U.S. firms and applying natural language processing (NLP) on 10-K reports, we have developed two types of COVID dictionaries (or COVID-related disclosure measurement tools): (a) overall COVID dictionary (count of all COVID-related words/phrases) and (b) contextual COVID-dictionary (count of COVID related words/phrases preceded or followed by positive, negative tones, or financial constraints words). Subsequently, we have validated both types of COVID dictionaries by investigating their association with corporate liquidity events (e.g., dividend payment, dividend change). We confirm that the overall COVID dictionary effectively predicts a firm’s liquidity event. We find similar results for contextual COVID dictionaries with a negative spin (i.e., COVID disclosures with a negative tone or an indication of financial constraints). Our results further show that better-governed firms (e.g., greater board independence, and more female directors) tend to have more COVID-related disclosures, despite the fact that more COVID-related disclosures suppress a firm’s market-based stock performance (e.g. Tobin’s Q). Our results suggest that better-governed firms prefer greater transparency, even if it may hurt their market performance in the short run.

## 1. Introduction

The global economy has experienced its worst crisis as a result of the COVID-19 pandemic since the Great Depression [1–4]. This uncertain economic environment has profoundly impacted firms’ operational activities and profitability around the world [5, 6]. Under such turbulent periods, firms’ shareholders and stakeholders anxiously await corporate communication to evaluate their investment positions [7]. One of the widely used channels for effective corporate information disclosure is the annual report (also known as the 10-K report), which helps reduce information asymmetry between a firm’s managers and shareholders—as well as other stakeholders—as highlighted by Leuz and Wysocki [8]. However, sharing negative development (such as the impacts of COVID on firm performance) through corporate disclosure could be very delicate [9]. Managers need to strike the right balance between transparency and containment of negative sentiments among market participants. Therefore, the question that we ask in these uncertain times is: how do companies disclose COVID-related information?

The tone of corporate disclosure at a time of turbulent economic conditions (e.g., COVID-19) tends to be conflicting in nature. On one hand, negative disclosure hampers firm performance (e.g., stock return) and hence discourages managers to disclose negative development [10, 11]. On the other hand, suppression of negative disclosure could increase the risk of litigation and attract the attention of regulatory authorities [12]. To that effect, it is interesting to examine how firms disclose firm-level information during COVID-19, mainly due to the inherent uncertainty associated with the COVID-19 pandemic. However, one of the major challenges to examining the corporate disclosure strategy in the face of a pandemic is that, to the best of our knowledge, there is no systematic measure to gauge the intensity of COVID-related disclosure [9]. Though Loughran and McDonald [13] presented an ad-hoc wordlist (consisting of 18 words or phrases) for COVID-19, it has two major limitations. First, the wordlist was developed quite subjectively at the early stage of the COVID-19 pandemic. Moreover, the COVID dictionary proposed by Loughran and McDonald [13] is not effective as they have not considered a versatile and comprehensive wordlist. Second, COVID-related disclosure can be contextual as 10-K reports discuss COVID-19 in different contexts (e.g., financial constraints, negative/ positive outlook, litigation) that have not been comprehensively addressed by the Loughran and McDonald [13] methodology.

In light of the above-mentioned limitations, this study develops a measure of COVID-19 disclosure i.e., the COVID dictionary using 3226 firms listed on S&P–it is based on corporate disclosure word frequency measure. Most importantly, we used 10-K reports to track different contexts around COVID-related words and thus also develop a contextual COVID dictionary. To that effect, we collected 10-K reports from the Edgar database for all the publicly traded companies in the US for the year 2020. Thereafter, we considered the textual content of the Management Discussion and Analysis (MDA) section of every 10-K report for generating two types of COVID dictionaries: (a) overall COVID-dictionary (count of all COVID-related words) and (b) contextual COVID-dictionary (count of COVID related words that are preceded or followed by positive, negative tone, and financial constraints words). The overall COVID-dictionary would allow us to examine the extent of COVID-related disclosure in MDA section. However, the firm management may use COVID-related disclosures in different contexts. In light of Loughran and McDonald (LM)’s and Bodnaruk, Loughran and McDonald (BLM)’s work, we additionally explore three different contextual COVID-related dictionary to investigate the impact of COVID-related disclosures at a more granular level. Accordingly, we have developed two sets of dictionaries: overall and contextual COVID-related dictionary. The methodology is explained in Section 3.

In order to make our wordlist well encompassing, we have employed Word2vec methodology to include all relevant synonyms from the annual report corpus. Subsequently, following Bodnaruk, Loughran [10] we validated both types of COVID dictionaries by examining their relationship with corporate liquidity events (e.g., dividend payment, dividend change). In line with Bodnaruk, Loughran [10], we find that our overall COVID dictionary (i.e. frequency of all COVID-related disclosures) effectively predicts a firm’s liquidity position and event (such as dividend payouts and dividend changes over the past year). We also find the relevance of our contextual COVID-related word frequency (i.e. dictionary). For instance, we find that COVID-related disclosure with a positive spin does not have any significant negative correlation with liquidity position or events. Whereas, COVID-related disclosure with a negative spin (when COVID-related words are used in conjecture with LM negative wordlist or BLM constraining wordlist), does have a significant negative association with liquidity position or events. It implies that in U.S. firms, management is generally transparent with their COVID-related disclosure, which reflects a firm’s anticipated liquidity positions.

Since COVID-related disclosure is quite useful to shareholders and market participants [14, 15], we subsequently explore which types of firms are more forthcoming in making COVID-related disclosures despite the risk of negative market reactions. As we discussed above, ideally well-governed firms are more likely to take initiatives to reduce information asymmetry, which would in turn help alleviate litigation risks [16, 17]. At the same time, in line with the prior studies [9, 18], we find that COVID-related disclosure (both overall and contextual), does have a significant negative association with Tobin’s Q. Interestingly, we find that firms with higher COVID-related disclosures are more careful with employee retention. It implies that the firms that are engaged in more self-disclosures are also proactive in containing negative influences on the organizational workforce.

The main contribution of our study is the creation and validation of overall and contextual COVID dictionaries to understand the plausible impacts of COVID on firm performance and operation. Only very few non-U.S. based studies have attempted to analyze COVID-related corporate disclosure so far [9]. For instance, using European firms’ annual reports, Bostan, Bunget [19] have examined how readability and uncertain tones in corporate disclosures have changed during the COVID era. Yet, this study does not focus on specific COVID-related disclosure. Another relevant study by Elmarzouky, Albitar [9] has used ‘COVID-19 Secure Guidelines’ published by the UK government to develop a COVID-related wordlist for UK firms and examine the correlation with annual report uncertainty. However, Elmarzouky, Albitar [9] wordlist is solely based on government guidelines and has not been contextualized with annual report contents. To the best of our knowledge, no study has conceptualized and validated a systematic measure to gauge the intensity of COVID-19 disclosure in the U.S. market. Further, we have developed various contextualized COVID-related dictionaries in order to provide a deeper insight to the shareholders and other market participants on firm-specific COVID-related developments. Accordingly, we examined how corporate governance structure influences COVID-related disclosures. To our knowledge, this is the first study to use a neural network-based word embedding model (i.e. Word2vec) that considers COVID-related semantics rooted in corporate disclosures (or 10-K reports). Rather than relying simply on a pre-determined set of terms (e.g. bag-of-words) or an entirely unsupervised approach (e.g. topic modeling using LDA), we employ a semi-supervised natural language processing (NLP) methodology [20, 21]. This research makes use of a semi-supervised approach, which begins the dictionary-building process with a predetermined set of seed words before proceeding to inductively collect information on COVID-related disclosures from the designated corpus (which in this case includes annual reports) [22].

The paper proceeds as follows. Section 2 presents the literature review; Section 3 presents the data and methodology, including a detailed dictionary-building process; Section 4 presents the results and relevant discussions; and Section 5 concludes the findings.

## 2. Literature review

### 2.1. Corporate disclosure and plausible impact

The term "corporate disclosure" is used to describe the process by which a firm documents its business activities in its financial statements in accordance with a set of generally accepted accounting principles and other reporting criteria [23]. Corporate disclosure’s objective is to make relevant data and information available to all stakeholders and market participants in a transparent manner [24–26]. Like transparency, tone of the corporate disclosure is quite important for market participants and stakeholders [27]. Behavioral theories posit that negative words have a greater influence than positive words–which highlights the importance of tone in corporate disclosure (e.g. annual reports) [28]. Therefore, managers prefer the optimistic “bag-of-words” while disclosing corporate-related information. According to Loughran and McDonald [27], managers are likely to use a positive tone in 10-K reports and such practices are negatively correlated with stock returns. In addition, Davis, Ge [29] have pointed out that managers can benefit from the chosen tone of corporate disclosure, especially when this information is more sensitive to stock prices.

It has been reported by Tan, Libby [30] that financial analysts’ reactions to disclosure vary depending on the manner in which it was disclosed; for example, if bad news were disclosed repeatedly, financial experts’ projections would be conservative. This demonstrates that market reactions are not always the same, regardless of the content of the information being given [24]. When choosing disclosure tactics in light of market reactions, some approaches are naturally more alluring to corporate managers than others. Companies that reveal bad news are more likely to disclose interest-related information in advance to avoid future litigation, as shown by Skinner, Marsh [31]. Financial reports, which are essential for making investment-related decisions, are widely available to the public. Since a plethora of studies are being conducted on quantitative reporting, investigating the subjective reporting preferences of business managers is poised to become a major area of inquiry. It is important to highlight that the subjective tone of corporate disclosure has a significant role in investment-related decisions, especially during unforeseen economic events (e.g., economic shock, unemployment rates, COVID-19) [3].

### 2.2. Implications of COVID-19 disclosure

The recent outbreak of the COVID-19 pandemic can be considered as a negative economic event because it has led to a worldwide recession [2, 19, 32]. As far as we understand, COVID-19 is the first pandemic to cause such widespread economic disruption in modern times [33, 34]. The first half of 2020 saw the biggest decline in real GDP since World War II, with the United States experiencing an 11% decline and Europe experiencing a 15% decline [3]. Most importantly, the global supply chain has been significantly disrupted due to the COVID-19 pandemic, subsequent widespread lockdown of cities and countries, and travel restrictions implemented by various governments globally [5]. As a result, business sectors like airline, clothing, consumer durables, vehicle manufacturing, electronics manufacturing, the hotel business, and financial services were all hit particularly hard [35]. Therefore, it is interesting to study how companies deal with the potential negative effect of the COVID-19 pandemic by communicating with investors and other interested parties through their annual disclosure [36]. Leuz and Wysocki [8] state that disclosures are an appropriate instrument for lowering the knowledge gap between management and the firm’s stakeholders, including its shareholders. Accordingly, in this study, attention is being directed to how organizations have reported their annual disclosure during the COVID-19 disruption and its impact on corporate policy and performance.

Recent business and management literature has discussed the COVID-19 pandemic and its impact on market reactions [9]. For instance, the findings of Erdem [37] show that COVID-19 has a deleterious effect on the market and increased volatility. Mazur, Dang [1] provide credence to the idea that high asymmetric volatility is linked with lower stock returns. They also emphasize that an uncertain economic environment results in higher levels of volatility [38, 39]. Goodell [40] offers theoretical perspectives on COVID-19 across financial markets, banking, and insurance, as well as government and public enterprises. The aforementioned studies on COVID-19 present how uncertainty affects the characteristics of business entities and their environments, prompting the inquiry as to whether or not the inclusion of information relevant to COVID-19 increases uncertainty in annual reporting. In these uncertain markets, COVID-related disclosure has become quite important for shareholders, stakeholders, and market participants.

The need of providing factual information and reporting amid times of uncertainty presents a significant challenge. Organizations across the globe have experienced the consequences of the recent COVID-19 pandemic. Therefore, the way "positive or negative news" was conveyed to the stakeholders through corporate disclosures is a matter of concern [9]. Some disclosure strategies are meant to obscure negative news, while others are intended to accentuate positive news. Therefore, the way information is projected plays a significant role on investors’ decisions, especially via the lens of ‘impression management’ techniques [41]. Wang and Xing [35] have shown that firms have used a low level of ‘uncertainty tone’ in 10-K reports during the COVID-19 pandemic period while announcing earning reports. One of the major challenges in COVID-related disclosure studies is the measurement of the COVID-related disclosure level itself. Only a few studies have focused on this important issue. Loughran and McDonald [13] presented an ad-hoc wordlist (consisting of 18 words or phrases) for COVID-19 disclosure. Our investigation shows that many of those words are not even presented in the annual report corpus. Elmarzouky, Albitar [9] have used ‘COVID-19 Secure Guidelines’ published by the UK government to develop a COVID-related wordlist for UK firms. However, since this wordlist was developed independent of the annual report content, it is likely that many relevant words could be excluded from the list.

### 2.3. Role of corporate governance in COVID-related disclosure

Information asymmetry is a fundamental contributor to the agency problem between the principal (shareholders) and agent (management) [42, 43]. Due to their day-to-day involvement in corporate activity, the manager may have access to information that shareholders and other key stakeholders may not [44, 45]. This information advantage may induce managers to take activities that benefit them,—for example, excessive compensation and empire-building at the expense of shareholders [46, 47]. Transparency and disclosure become crucial to lessen the information asymmetry in these situations [42, 48]. Annual reports are management’s tool to communicate with shareholders and stakeholders regarding performance, which is becoming increasingly crucial for market participants [15] during economic shocks (e.g., COVID-19) [3]. Due to the most recent global supply chain disruption, investors are more concerned about COVID-related disclosure while making investment decisions. Literature has emphasized that transparent COVID-related corporate disclosure reduces information asymmetry but creates more uncertainty in the annual report [9]. In addition, the disclosure of COVID-related information is a strategic decision and depends on the selection and motives of the management and directors. Earlier studies have shown that the level of corporate disclosure is hugely sensitive to corporate governance [17, 42, 49, 50].

The function of an independent director becomes critical as it is the representative of shareholders, other stakeholders on the board. Further, it is responsible for protecting the shareholders’ interests [51, 52]. Independent directors are completely independent of management and have no personal interests in the firm. By virtue of their independence, independent directors enable greater supervision of management [53] and enhance the efficacy of the board [54]. Independent directors also assist in controlling agency expenses [55]. They are not only accountable to shareholders, but also ensure the well-being of other stakeholders [56]. Moreover, they motivate companies to disclose high-quality, transparent information [17]. Previous research indicates that a greater percentage of independent directors in a company boosts its emphasis on quality and transparent disclosure [57, 58]. In order to mitigate problems associated with corporate misconduct, such as manipulated corporate disclosure and concealment of COVID-related disclosure, boards must include independent directors [3, 59–61]. Considering the above arguments, it might be argued that board independence facilitates transparent and quality COVID-related disclosure.

Gender diversity among board members is another critical factor influencing the accuracy of corporate disclosure [62, 63]. Gender socialization theory implies that women and men have different perspectives on moral and ethical behavior [64, 65]. It might be argued, as Gilligan [66] does in her ‘ethics of care’, that women gravitate toward ethical behavior more so than men. Furthermore, the ethics of care theory suggests that women’s moral growth makes them more capable than men of meeting the needs of others [67]. Existing empirical studies demonstrate a correlation between female board membership and corporate disclosure [68–70]. Harjoto and Rossi [71], have demonstrated empirically that the number of female board members is positively correlated with disclosure. Moreover, a meta-analysis reveals that the presence of women in the boardroom is positively associated with improved performance [72]. Therefore, it is plausible to assert that female directors can promote more efficient and transparent COVID-related disclosure, given the aforementioned justifications.

## 3. Data and methodology

### 3.1. Data and dictionary building

#### 3.1.1. Sample collection

This study focuses on S&P-listed companies. We began with a sample of 7383 firms. However, after excluding firms with inconsistent and missing data, the final sample size is 3226 firms. Further, we look into firms’ annual reports (also known as 10-K reports) to analyze overall and contextual (i.e. presented in light of positive/ negative tones, and financial constraints) COVID-related disclosures. To that effect, we have used parameters considered by Loughran and McDonald [13] to identify positive/negative, and financial constraints attribute related to COVID. Subsequently, using Edgar database, we have collected 10-K reports of U.S. publicly listed firms for the fiscal year 2020. These reports contain firm-related information for fiscal year 2020, and are generally published in year 2021. Subsequently, we have examined the Management’s Discussion and Analysis (MDA) section of each 10-K report to create overall and contextual COVID-related wordlist(s). We have used text mining techniques and cleaned up the MDA corpus using various data pre-processing techniques—which is explained in the next sub-section (3.1.2).

#### 3.1.2. Data pre-processing

In this study, we have processed the text at multiple stages with the help of Spacy (version 2.3.5)—*a free*, *open-source Natural Language Processing library for Python*, Stanza (version 1.1.1), Gensim (version 3.8.3), and Regular Expression package (version 2020.11.13) toolkits [73]. We have briefly discussed these stages below:

- Pre-processing sentences: Regex has been used to eliminate “E-mails, URLs, punctuations, new line characters, single characters, digits (i.e., numbers), and extra spaces” [22, 74].
- Named Entity Recognition (NER): Utilizing Spacy, we implemented Named Entity Recognition (NER) to identify locations, people, and organizations within the text. Afterward, we removed the detected Named Entities from the corpus [22].
- Lemmatization: The lemmatization task has been performed by using the Spacy library. Lemmatization of a word returns its root form. For instance, “uncertainties to uncertainty”, “encouraging to encourage”, “improved to improve”, “opportunities to opportunity”, etc.
- Stop words and Tokenization: Following the elimination of stop words, we performed tokenization to break the text into its individual tokens. The Gensim library’s ’simple_preprocess’ function was used to tokenize each sentence and delete tokens that had fewer than three characters. It is essential to remove the stop words before employing n-gram models (or, phraser model), because this allows us to recognize more corpus-specific phrases with precision. Otherwise, the phraser model would create phrases that are less meaningful.

#### 3.1.3. Word embedding and Word2vec model

*3*.*1*.*3*.*1*. *Word embedding*. Word embedding is a way of representing words by assigning them numerical values in a vector space of dimension n, which encapsulates the semantic meaning of the word. This is known as word’s “distributed representation”, as the semantics of a word is spread across all the dimensions in the vector. The word embedding of a word allows us to assess the relationship between two words by measuring the cosine similarity of their respective feature vectors. Consequently, the feature vectors of a seed word and every other word in the corpus can be compared to generate a larger set of words and phrases that describe a particular theme/concept. Further, this expanded set of words can be used to capture the extent of that theme in the corpus.

*3*.*1*.*3*.*2*. *Word2vec model*. Word2vec is a two-layer neural network that generates low-dimensional, dense representations of words that contains information of the meaning and context of a word. The Word2vec model is trained to learn the meanings of words by taking a large corpus of words as input and generating a vector space of several hundred dimensions, with each word in the corpus being assigned a vector. In simple terms, Word2vec attempts to predict a word given the adjacent words and learns the numerical representation of the word in the process. Words that appear in similar contexts across the corpus are clustered together in the vector space.

Word2vec is a computationally efficient predictive model for learning word embeddings from raw text. It uses neural network architecture with random parameters and adjusts its parameters (or weights) via a backpropagation algorithm in order to reduce prediction errors (i.e., predicting the context words of the specific or focal word). In our analysis, the Word2vec model will not be used to predict the neighboring words; rather, the information it has gained during the learning process will be acquired. This includes taking the hidden weights which become an “effective feature vector representation of the word when learning is completed after a number of iterations through the documents” Li, Mai [22], p. 13. This results in vector space of specific dimension (ranging between 50–500) whereby vector corresponding to every unique word will be present. As mentioned earlier, resulting vectors signify the meaning and the connection between a central word/phrase and its neighbors.

#### 3.1.4. Implementation of the Word2vec model

The Genism toolkit was employed in the pre-processing and parsing of the corpus text for the purpose of training the Word2vec model with the MDA section of 10-K reports released in 2020 (3,927 annual reports). The Skip-gram and CBOW models, as defined by Mikolov, Sutskever [75], were generated manually with the help of deep learning tools such as TensorFlow and PyTorch. In order to implement the Word2vec model, we had to consider several hyperparameters—for example, ignoring words whose frequency of occurrence in the corpus is lower than five, the dimensions of the distributed representation of words were set to 300, and considering two words as neighbors when they appear within five words of a sentence [22]. The Word2vec model uses neighbor words to learn the context of the focal word. After the training process, each unique token in the Word2vec model was given a 300-dimensional vector that encapsulated the meaning of each word or phrase.

#### 3.1.5. Developing a representative COVID dictionary

Loughran and McDonald [13] presented an ad-hoc wordlist (consisting of 18 words or phrases) for Coronavirus/COVID. However, as the wordlist was developed quite subjectively at the early stage of the pandemic, it appears to be rather less representative of the pandemic theme in narrative disclosures. To that effect, we carried a preliminary test to check the presence of Loughran and McDonald’s (hereafter, LM) COVID dictionary words and phrases in our MDA corpus. We found that out of 18 LM COVID words/phrases, only six were present in the corpus (namely, *pandemic*, *epidemic*, *contagious_disease*, *and infectious_disease*, *mers*, *ebola)* (Please refer to S1 Table, Panel 1B).

To obtain a more representative COVID dictionary, we obtain synonyms of the LM COVID dictionary words from the MDA corpus. To that effect, we utilized word vectors generated by the Word2vec model (explained in the previous section) to compute the cosine similarity between LM COVID dictionary words and every other word of the MDA corpus (S1 Table, Panel 1C). We followed a two-step process to eliminate the irrelevant words from the LM COVID dictionary and come up with additional COVID-related words. First, we examined the synonyms of the LM COVID dictionary words and eliminated those words whose synonyms were not representative of the theme associated with the word. In the process, we eliminated two seed words (*‘mers’*, *‘ebola’*) from LM COVID dictionary (S1 Table, Panel 1D). Second, using the synonyms of the remaining LM COVID dictionary words (namely, *pandemic*, *epidemic*, *contagious_disease*, *and infectious_disease*) we identified a set of additional COVID-related words/phrases (S1 Table, Panel 2A). As described by Li, Mai [22], like before, we verify the selection of these additional COVID words by examining their synonyms (S1 Table, Panel 2B).

Further, we investigated the MDA corpus and checked the bigrams that include COVID/coronavirus as one of the constituents of bigram. This allowed us to include COVID and coronavirus-related bigrams in our dictionary (such as *covid_pandemic*, *coronavirus_pandemic*). Accordingly, we have only included relevant bigrams in the dictionary after investigating the synonyms of selected words from the corpus (please refer to S1 Table, Panel 2A). Examining the synonyms of a word allowed us to internally validate the proposed COVID dictionary.

To test the robustness of the procedure that we followed to internally validate our dictionary words, we examined synonyms of a few randomly selected words that are not directly relevant to COVID. As expected, their synonyms are not representative of the COVID phenomenon (this process is similar to the examination of the relevance of words *‘mers’* and *‘ebola’* of the LM wordlist).

Finally, we combined the refined sets of both included words from the LM dictionary and new COVID-related words to come up with the final COVID dictionary (S1 Table, Panel 3A). A flowchart has been included in S2 Table that summarizes the methodology employed to create the overall COVID dictionary. Note that, in this study, we also generated n-grams to populate the COVID dictionary with more versatile words which makes the COVID dictionary more effective. Further, we split all n-grams—except for the ones which belong to the COVID dictionary—as LM dictionaries (namely, positive, and negative–these word lists are available at https://sraf.nd.edu/loughranmcdonald-master-dictionary/) and BLM dictionary (financial constraints) only consist of unigrams. This is because unless we split the n-grams, it will not reflect the true count of dictionary words. The refined list of COVID seed words/ phrases includes the following:

’coronavirus’, ’coronavirus_pandemic’, ’pandemic’, ’COVID_pandemic’, ’COVID’, ’novel_coronavirus’, ’novel_strain_coronavirus’, ’COVID_outbreak’, ’resurgence_COVID’, ’health_crisis’, ’public_health_crisis’, ’outbreak’, ’novel_strain’, ’novel_coronavirus_disease’, ’coronavirus_disease’, ’epidemic’, ’contagious_disease’, ’infectious_disease’

#### 3.1.6. Generating contextual COVID list

The above COVID wordlist will allow us to check the extent of COVID-related disclosure in the MDA section. However, the firm management may use COVID-related disclosures in a different context. In light of LM’s and BLM’s work, we explore three different contextual COVID-related dictionaries to investigate the impact of COVID-related disclosures at a more granular level. These three themes are COVID words in a *positive context*, *negative context*, and *financial constraints context*. To track the impact of COVID words in different contexts (Positive, Negative or Financial Constraints), we generated the signal using the WINDOW algorithm discussed below.

In the WINDOW algorithm, unlike plain term frequency, we placed constraints on when to tally up the count of the LM or BLM dictionary words. We tagged the COVID dictionary words in the corpus and applied the condition to update the Positive, Negative or Financial Constraints word count (based on LM or BLM dictionary), only if the word is present within the 25-word window of the COVID dictionary words (as discussed in section 3.1.5). This algorithm allowed us to capture the extent of different contexts (specifically, positive, negative, and financial constraints) in relation to disclosures regarding COVID-19.

Note that in our COVID dictionary, we have included n-grams. To use the COVID and LM dictionaries effectively, we first trained the Phraser model and included n-grams in the corpus. However, since LM dictionaries consist of only unigrams, we only keep the n-grams that are present in the COVID dictionary and split the remaining ones to get precise scores. For more MDA 2020 related examples, please refer to S1 File.

### 3.2. Validation of COVID dictionary and impact of corporate governance

In order to examine the validity of the COVID dictionary and the impact of corporate governance on COVID-related disclosures, we employ several multiple regression analyses. Specifically, we examine (a) the relationship between COVID-related disclosure (proxied by the frequency of COVID-related disclosure, contextual COVID-related positive word frequency, contextual COVID-related negative word frequency, contextual COVID-related financial constraints word frequency) and corporate liquidity events (e.g., dividend payment, dividend change), and (b) the relationship between corporate governance characteristics (measured by board independence, and female directors) and COVID-related disclosure. To ensure the robustness of our regression results, we control for the industry effect and adjust for plausible heteroskedasticity in our sample [76]. We have collected firm-specific and financial information from the COMPUSTAT database and governance (i.e. Board) data from the BOARDEX database.

#### 3.2.1. COVID-related disclosure and corporate liquidity events

To investigate the association between COVID-related disclosure and corporate liquidity events, we estimate Eq 1. Table 1 presents detailed information on variables used in the following regression model. We used the OLS methodology for all regression models. In the regression models, we control for ’Fama-French 48-category’ (FF48) industry effects. We further control for heteroscedasticity and report robust t-statistics [77].


$$\begin{array}{l} {\left(Corporate\;liquidity\;events\right)}_{it}=\beta_{0}+\beta_{1}\times {\left(frequency\;of\;COVID‐related\;disclosure\right)}_{it}+\\ \beta_{2}\times {\left(Contextual\;COVID‐related\;positive\;word\;frequency\right)}_{it}+\beta_{3}\times (Contextual\;COVID‐related\\ negative\;word\;frequency{)}_{it}+\beta_{4}\times (Contextual\;COVID‐related\;financial\;constraints\;word\\ frequency{)}_{it}+\beta_{5}\times {\left(Herfidahl\;index\right)}_{it}+\beta_{6}\times {\left(Long‐term\;debt\;to\;assets\right)}_{it}+\beta_{7}\times {\left(Firm\;size\right)}_{it}+\\ \beta_{8}\times {\left(R\&D\;expenditure\right)}_{it}+\beta_{9}\times {\left(Return\;on\;assets\right)}_{it}+Industry\;effect+\varepsilon_{it} \end{array}$$ (Eq 1)

Where corporate liquidity events are proxied by dividend payment and change in dividend payment.

**Table 1:** Variable description.

Variable	Description/Calculation
Dividend payments	Highlights the distribution of firm’s profit to its shareholders. Measured as total dividend divided by total assets.
Frequency of COVID-related disclosure	Number of COVID-related words in 10-K reports.
Contextual COVID-related positive word frequency	Extent of COVID-related positive sentiment embedded in 10-K reports.
Contextual COVID-related negative word frequency	Extent of COVID-related negative sentiment embedded in 10-K reports.
Contextual COVID-related financial constraints word frequency	Extent of COVID-related financially constraining sentiment embedded in 10-K reports.
Herfindahl index	A measure of market concentration, based on annual revenue of firms in an industry.
Long-term debt to assets	It highlights the percentage of firm’s total assets financed by its long-term debts. It is also known as coverage or solvency ratio.
Firm size	Log of the market value of equity
R&D expenditure	A firm’s investment in research and development (R&D) program. Measured as R&D expenditure by sales.
Return on assets	It highlights a firm’s profitability in relation to total assets. It is the ratio of net income to total assets.

#### 3.2.2. Corporate governance characteristics and COVID-related disclosure

Past studies have argued that the level of corporate disclosure is significantly influenced by the structure of corporate governance [42, 49, 78]. We extend their arguments by examining the impact of strong corporate governance on COVID-related disclosure using Eq 2.


$$\begin{array}{l} {\left(COVID‐related\;disclosure\right)}_{it}=\beta_{0}+\beta_{1}\times {\left(Board\;independence\%\right)}_{it}+\beta_{2}\times (Female\\ director\%{)}_{it}+\beta_{3}\times {\left(Herfindahl\;index\right)}_{it}+\beta_{4}\times {\left(Long‐term\;debt\;to\;assets\right)}_{it}+\beta_{5}\times {\left(Firm\;size\right)}_{it}+\\ \beta_{6}\times {\left(Total\;dividend\;dummy\right)}_{it}+\beta_{7}\times {\left(R\&D\;expenditure\right)}_{it}+\beta_{8}\times {\left(Return\;on\;assets\right)}_{it}+Industry\\ effect+\varepsilon_{it} \end{array}$$ (Eq 2)

Where COVID-related disclosure is measured by frequency of COVID-related disclosure, contextual COVID-related positive word frequency, contextual COVID-related negative word frequency, and contextual COVID-related financial constraints word frequency.

### 3.3. Descriptive statistics and correlation matrix

Table 2 shows the industry distribution of the firms included in our final sample. Further, we have presented descriptive statistics of the key variables used in the current study in Table 3. Table 4 presents the correlation matrix of our variables. The univariate results show that there is a significant negative correlation between the overall *COVID dictionary* (e.g., *frequency of COVID-related disclosure)* and *dividend payment*. It provides a preliminary indication that higher COVID-related disclosure leads to lower dividend payments. However, univariate analysis can be misleading, as they overlook any confounding effects [79].

**Table 2:** Industry-wise sample distribution.

FF48 industry category number	FF48 industry category name	Number of firms	Percent	Average proportion of sales
1	Agriculture	84	2.6	0.016665
2	Food Products	212	6.57	0.031007
3	Candy & Soda	425	13.17	0.031976
4	Beer & Liquor	93	2.88	0.037441
5	Tobacco Products	77	2.39	0.040382
6	Recreation	60	1.86	0.044483
7	Entertainment	242	7.5	0.046431
8	Printing and Publishing	415	12.86	0.047897
9	Consumer Goods	27	0.84	0.048176
10	Apparel	52	1.61	0.049515
11	Healthcare	43	1.33	0.050556
12	Medical Equipment	69	2.14	0.055845
13	Pharmaceutical Products	84	2.6	0.056518
14	Chemicals	126	3.91	0.056759
15	Rubber and Plastic Products	59	1.83	0.060509
16	Textiles	120	3.72	0.065768
17	Construction Materials	39	1.21	0.070184
18	Construction	128	3.97	0.075681
19	Steel Works Etc	88	2.73	0.076777
20	Fabricated Products	25	0.77	0.078354
21	Machinery	27	0.84	0.082479
22	Electrical Equipment	8	0.25	0.083908
23	Automobiles and Trucks	63	1.95	0.084322
24	Aircraft	50	1.55	0.084921
25	Shipbuilding, Railroad Equipment	51	1.58	0.091601
26	Defense	133	4.12	0.096529
27	Precious Metals	38	1.18	0.101353
28	Non-Metallic and Industrial Metal Mining	37	1.15	0.112301
29	Coal	10	0.31	0.113424
30	Petroleum and Natural Gas	12	0.37	0.123769
31	Utilities	13	0.4	0.127602
32	Communication	35	1.08	0.13024
33	Personal Services	63	1.95	0.15351
34	Business Services	23	0.71	0.155951
35	Computers	21	0.65	0.163241
36	Electronic Equipment	13	0.4	0.177165
37	Measuring and Control Equipment	9	0.28	0.191552
38	Business Supplies	7	0.22	0.194584
39	Shipping Containers	15	0.46	0.199744
40	Transportation	4	0.12	0.216387
41	Wholesale	9	0.28	0.23059
42	Retail	36	1.12	0.232579
43	Restaurants, Hotels, Motels	3	0.09	0.259573
44	Banking	9	0.28	0.315093
45	Insurance	53	1.64	0.338912
46	Real Estate	7	0.22	0.393796
47	Trading	5	0.15	0.562372
48	Others	4	0.12	0.808254

**Table 3:** Descriptive analysis.

Variable	Observations	Mean	Std. Dev.	Min	Max
Dividend payments	3226	0.0214926	0.0453824	0	0.2766621
Frequency of COVID-related disclosure	3226	4.237462	2.61938	0	12.20339
Contextual COVID-related positive word frequency	3226	1.710241	1.473701	0	6.692694
Contextual COVID-related negative word frequency	3226	5.318882	3.732315	0	16.97497
Contextual COVID-related financial constraints word frequency	3226	1.65849	1.480847	0	6.680919
Herfindahl index	3226	0.0712691	0.0649835	0.016665	0.8082542
Long-term debt to assets	3226	0.2772122	0.2535747	0	1.418853
Firm size	3226	6.947227	2.227266	0.260728	11.84363
R&D expenditure	3226	1.509341	8.732084	0	77.93104
Return on assets	3226	-0.1016487	0.4579364	-11.48521	0.3953488

**Table 4:** Correlation matrix.

	Variables	[1]	[2]	[3]	[4]	[5]	[6]	[7]	[8]	[9]	[10]
[1]	Dividend payments	1									
[2]	Frequency of COVID related disclosure	-0.0353*	1								
[3]	Contextual COVID related positive word frequency	0.0111	0.632***	1							
[4]	Contextual COVID related negative word frequency	-0.0436*	0.788***	0.544***	1						
[5]	Contextual COVID related financial constraints word frequency	-0.0353*	0.673***	0.414***	0.664***	1					
[6]	Herfindahl index	0.0213	0.0849***	0.0594***	0.0530**	0.0650***	1				
[7]	Long-term debt to assets	0.0786***	0.0667***	0.0590***	0.0530**	0.131***	0.0112	1			
[8]	Firm size	0.215***	0.0242	0.114***	-0.0126	-0.0205	-0.0204	0.129***	1		
[9]	R&D expenditure	-0.0694***	-0.0869***	-0.0867***	-0.0963***	-0.0650***	-0.0479**	-0.113***	-0.0877***	1	
[10]	Return on assets	0.156***	0.0740***	0.0958***	0.0613***	0.0434*	0.0244	-0.0170	0.287***	-0.198***	1

*** p<0.01

** p<0.05

* p<0.10

## 4. Results and discussion

We organize this section as follows. As per the main objective of the paper, first, we present relevant results to validate our COVID dictionaries. More specifically, we examine the relation between our COVID dictionaries and liquidity events. Subsequently, we present and discuss the results that highlight the impact of corporate governance on COVID-related disclosures. Finally, in order to get a holistic perspective, we further examine the relation between (a) COVID-related disclosures and a firm’s market-based stock performance (Tobin’s Q), and (b) COVID-related disclosures and a firm’s employee turnover.

### 4.1. COVID-related disclosure and dividend payment

This section examines the association between the overall COVID dictionary (proxied by the frequency of COVID-related disclosure), contextual COVID dictionary (proxied by COVID-related positive word frequency, contextual COVID-related negative word frequency, contextual COVID-related financial constraints word frequency), and dividend payment. Results are presented in Table 5. Model 1 shows the impact of the *frequency of COVID-related disclosure* on dividend payments in the presence of other control variables. We find that the coefficient on the *frequency of COVID-related disclosure* is negative (-0.001) and significant at the 1 percentile level. It implies that a high frequency of COVID-related words leads to lower dividend payouts. In line with Bodnaruk, Loughran [10] findings, we can infer that more COVID words signify uncertainty within the firm, and therefore, companies are more likely to decrease their dividends. Model 2 (Table 5) shows the relationship between *contextual COVID-related positive word frequency* and dividend payouts. The coefficient on *contextual COVID-related positive word frequency* is negative (-0.001) and insignificant. It suggests that a positive spin on the COVID-related disclosure means companies are better able to deal with COVID-related uncertainties and therefore, unlikely to affect dividend payments. In Model 3 and Model 4 (Table 5), the coefficient on *contextual COVID-related negative word frequency* (-0.001) and *contextual COVID-related financial constraints word frequency (0*.*002)* are statistically significant at the 1 percentile level. It highlights that as the frequency of negative and financially constraining words in 10-K reports rises, the likelihood of a reduction in dividend payment increases [9].

**Table 5:** Relationship between COVID-related disclosure and dividend payment.

	DV: Dividend payouts			
Variables	Model (1)	Model (2)	Model (3)	Model (4)
Frequency of COVID-related disclosure	-0.001***			
	(-2.985)			
Contextual COVID-related positive word frequency		-0.001		
		(-1.561)		
Contextual COVID-related negative word frequency			-0.001***	
			(-3.007)	
Contextual COVID-related financial constraints word frequency				-0.002***
				(-3.545)
Herfindahl index	-0.103***	-0.104***	-0.105***	-0.103***
	(-5.279)	(-5.341)	(-5.380)	(-5.354)
Long-term debt to assets	0.004	0.003	0.004	0.004
	(0.727)	(0.671)	(0.732)	(0.810)
Firm size	0.004***	0.004***	0.004***	0.003***
	(8.997)	(9.171)	(8.952)	(8.824)
R&D expenditure	-0.000	-0.000	-0.000	-0.000
	(-0.515)	(-0.421)	(-0.471)	(-0.418)
Return on assets	0.008**	0.008**	0.008**	0.008**
	(2.391)	(2.342)	(2.367)	(2.368)
Constant	0.024***	0.022***	0.025***	0.024***
	(3.480)	(3.176)	(3.561)	(3.401)
Industry effect	Yes	Yes	Yes	Yes
Observations	3,226	3,226	3,226	3,226
R-squared	0.117	0.115	0.117	0.118

*** p<0.01

** p<0.05

* p<0.10 (Robust t-statistics in parentheses)

### 4.2. COVID-related disclosure and net change in dividend payment

This section has investigated the relationship between the overall COVID dictionary (proxied by frequency of COVID-related disclosure), contextual COVID dictionary (proxied by COVID-related positive word frequency, contextual COVID-related negative word frequency, and contextual COVID-related financial constraints word frequency), and net change in dividend payment. This is illustrated in Table 6. In the presence of control variables, Model 1 demonstrates the impact of the *frequency of COVID-related disclosure* on the net change in dividend payments. The coefficient on the *frequency of COVID-related disclosure* (-0.005) is statistically significant at the 1 percentile level. It means that a high frequency of COVID-related words reduces the possibility that dividend payments will increase [10]. The coefficients on *contextual COVID-related positive word frequency* (-0.007), *contextual COVID-related negative word frequency* (-0.004), and *contextual COVID-related financial constraints word frequency* (0.009) are statistically significant at the 1 percentile level in Models 2, 3, and 4 (Table 6). It emphasizes that as the frequency of positive, negative, and financially constraining words in 10-K filings increases, the likelihood of a dividend increase lowers.

**Table 6:** Relationship between COVID-related disclosure and net change in dividend payment.

	DV: Change in dividend payouts			
Variables	Model (1)	Model (2)	Model (3)	Model (4)
Frequency of COVID-related disclosure	-0.005***			
	(-3.461)			
Contextual COVID-related positive word frequency		-0.007***		
		(-3.605)		
Contextual COVID-related negative word frequency			-0.004***	
			(-4.523)	
Contextual COVID-related financial constraints word frequency				-0.009***
				(-4.285)
Herfindahl index	-0.100**	-0.109***	-0.116***	-0.099**
	(-2.400)	(-2.717)	(-2.659)	(-2.336)
Long-term debt to assets	-0.058***	-0.060***	-0.058***	-0.057***
	(-2.948)	(-2.987)	(-2.929)	(-2.887)
Firm size	0.006***	0.007***	0.006***	0.006***
	(4.201)	(4.402)	(4.117)	(4.088)
R&D expenditure	0.002	0.002	0.002	0.002
	(1.205)	(1.213)	(1.208)	(1.229)
Return on assets	-0.005	-0.006	-0.006	-0.006
	(-0.659)	(-0.720)	(-0.730)	(-0.724)
Constant	0.019	0.007	0.024	0.014
	(1.295)	(0.545)	(1.626)	(1.001)
Industry effect	Yes	Yes	Yes	Yes
Observations	3,209	3,209	3,209	3,209
R-squared	0.045	0.044	0.046	0.045

*** p<0.01

** p<0.05

* p<0.10 (Robust t-statistics in parentheses)

### 4.3. Corporate governance and COVID-related disclosure

This section investigates the impact of board characteristics on overall COVID disclosure and contextual COVID disclosure. Model 1, and Model 2 (Table 7) present the relationship between the percentage of board independence, percentage of female directors, and overall COVID dictionary. The coefficients on the *percentage of board independence* (0.922), and *percentage of female directors* (1.463) are significant at 1 percentile level. It suggests that firms with more independent and female directors are likely to disclose more COVID-related information. The findings are consistent with past studies [9, 80, 81], suggesting that stronger governance promotes higher transparency and greater COVID-related disclosure.

**Table 7:** Relationship between governance characteristics and frequency of COVID-related disclosure.

	DV: Frequency of COVID-related disclosure	
Variables	Model (1)	Model (2)
Board independence%	0.922***	
	(2.769)	
Female director%		1.463***
		(3.140)
Herfindahl index	-1.034	-1.220
	(-0.318)	(-0.376)
Long-term debt to assets	0.398*	0.386*
	(1.942)	(1.887)
Firm size	-0.056**	-0.067**
	(-2.040)	(-2.396)
Total dividend dummy	0.163	0.152
	(1.406)	(1.319)
R&D expenditure	-0.016***	-0.016***
	(-2.677)	(-2.681)
Return on assets	0.160	0.189
	(0.839)	(0.991)
Constant	3.106***	3.602***
	(4.090)	(4.848)
Industry effect	Yes	Yes
Observations	2,954	2,954
R-squared	0.104	0.106

*** p<0.01

** p<0.05

* p<0.10 (Robust t-statistics in parentheses)

Subsequently, we examined how corporate governance impacts contextual COVID disclosure (proxied by COVID-related positive word frequency, contextual COVID-related negative word frequency, and contextual COVID-related financial constraints word frequency), and the results are presented in Tables 7–10. Table 8 (Model 1-Model 2) shows the relationship between the percentage of board independence, percentage of female directors, and COVID-related positive word frequency. *Ex-ante*, it is not clear how corporate governance would impact the tone of COVID-related disclosures [17, 42]. There is a possibility that the presence of a stronger governance structure would encourage firm managers to present business prospects during the COVID crisis more positively [9]. On the other hand, stronger governance may insist on presenting a more cautionary picture of firm prospects (i.e. less positive view) during the COVID era. Our results show that the coefficients on *the percentage of board independence* (0.695) and *percentage of female directors* (0.785) are significant at the 1 percentile level. It highlights that corporate disclosure is likely to have a more positive tone amid the COVID-19 pandemic if there is a high level of board independence and female directors.

**Table 8:** Relationship between board-level characteristics and contextual COVID-related positive disclosure.

	DV: Contextual COVID-related positive word frequency	
Variables	Model (1)	Model (2)
Board independence%	0.695***	
	(3.717)	
Female director%		0.785***
		(3.269)
Herfindahl index	-1.853*	-1.997*
	(-1.719)	(-1.908)
Long-term debt to assets	0.046	0.040
	(0.425)	(0.370)
Firm size	0.029*	0.028*
	(1.912)	(1.764)
Total dividend dummy	0.200***	0.194***
	(3.133)	(3.034)
R&D expenditure	-0.008***	-0.008***
	(-3.203)	(-3.230)
Return on assets	0.120	0.135
	(1.349)	(1.510)
Constant	0.572*	0.950***
	(1.826)	(3.244)
Industry effect	Yes	Yes
Observations	2,954	2,954
R-squared	0.124	0.124

*** p<0.01

** p<0.05

* p<0.10 (Robust t-statistics in parentheses)

**Table 9:** Relationship between board-level characteristics and contextual COVID-related negative disclosure.

	DV: Contextual COVID-related negative word frequency	
Variables	Model (1)	Model (2)
Board independence%	1.300***	
	(2.641)	
Female director%		2.057***
		(3.284)
Herfindahl index	-4.869	-5.132
	(-1.088)	(-1.152)
Long-term debt to assets	0.575*	0.558*
	(1.873)	(1.825)
Firm size	-0.141***	-0.157***
	(-3.528)	(-3.838)
Total dividend dummy	0.664***	0.649***
	(3.972)	(3.892)
R&D expenditure	-0.022***	-0.022***
	(-3.161)	(-3.175)
Return on assets	-0.253	-0.211
	(-0.809)	(-0.676)
Constant	5.050***	5.749***
	(4.366)	(5.148)
Industry effect	Yes	Yes
Observations	2,954	2,954
R-squared	0.099	0.101

*** p<0.01

** p<0.05

* p<0.10 (Robust t-statistics in parentheses)

**Table 10:** Relationship between board-level characteristics and contextual COVID-related financial constraint disclosure.

	DV: Contextual COVID-related financial constraints word frequency	
Variables	Model (1)	Model (2)
Board independence%	0.171	
	(0.855)	
Female director%		0.417
		(1.640)
Herfindahl index	-0.195	-0.228
	(-0.133)	(-0.156)
Long-term debt to assets	0.467***	0.464***
	(3.689)	(3.667)
Firm size	-0.061***	-0.066***
	(-4.040)	(-4.263)
Total dividend dummy	0.134**	0.131**
	(1.997)	(1.966)
R&D expenditure	-0.006*	-0.005*
	(-1.823)	(-1.801)
Return on assets	0.081	0.090
	(0.700)	(0.777)
Constant	1.378***	1.469***
	(3.689)	(4.049)
Industry effect	Yes	Yes
Observations	2,954	2,954
R-squared	0.116	0.116

*** p<0.01

** p<0.05

* p<0.10 (Robust t-statistics in parentheses)

Table 9 (Model 1-Model 2) shows the relationship between the percentage of board independence, percentage of female directors, and COVID-related negative word frequency. The results show that the coefficient on the *percentage of board independence* (1.3), and percentage of female directors (2.057), is significant at the 1 percentile level. These findings state that in the presence of more independent and female directors, corporate disclosure is likely to contain negative tones amid the economic and supply chain disruption caused by the COVID-19 pandemic [17, 44]. Therefore, we can construe that stronger governance enables firms to disclose more ‘bad news’ in a way to seamlessly share information with shareholders and other stakeholders, thus reducing information asymmetry [9]. Taken together, Tables 7–9 present some interesting results. These results highlight that stronger governance, in general, facilitates greater COVID-related information disclosures (overall, positive or negative). This is consistent with the view that firms with better governance tend to have better disclosure and lower information asymmetry.

Table 10 (Model 1-Model 2) shows the relationship between the percentage of board independence, percentage of female directors, and COVID-related financial constraint word frequency. The results show that the coefficients on the *percentage of board independence* (0.171) and *percentage of female directors* (0.417) are insignificant. It implies that corporate governance does not have much influence in disclosing financially constraining news at the time of COVID-19.

### 4.4. Additional analysis

#### 4.4.1. COVID-related disclosure and market-based financial performance

In Table 11 (Model 1 –Model 4), we have presented the relationship between COVID-related disclosure and Tobin’s Q. Tobin’s Q is a widely used measure of market-based performance [82–87]. In line with Manchiraju and Rajgopal [82], Tobin’s q is calculated as the sum of total assets and market value of equity less common book equity, divided by total assets. We observe that the coefficient on the *frequency of COVID-related disclosure* (-0.079), *contextual COVID-related positive word frequency* (-0.186), *contextual COVID-related negative word frequency* (-0.114), and *contextual COVID-related financial constraints word frequency* (-0.183) is significant at the 1 percentile level. It is in line with the observation presented in prior studies that more discussion on uncertain events (e.g., COVID) hampers a firm’s market-based financial performance, such as Tobin’s Q [9, 27].

**Table 11:** Relationship between COVID-related disclosure and Tobin’s Q.

	DV: Tobin’s Q			
Variables	Model (1)	Model (2)	Model (3)	Model (4)
Frequency of COVID-related disclosure	-0.079***			
	(-2.703)			
Contextual COVID-related positive word frequency		-0.186***		
		(-4.252)		
Contextual COVID-related negative word frequency			-0.114***	
			(-6.571)	
Contextual COVID-related financial constraints word frequency				-0.183***
				(-3.827)
Herfindahl index	-2.143	-2.418	-2.621*	-2.139
	(-1.420)	(-1.617)	(-1.814)	(-1.511)
Long-term debt to assets	-0.657	-0.663	-0.621	-0.613
	(-1.188)	(-1.203)	(-1.131)	(-1.110)
Firm size	0.444***	0.455***	0.435***	0.439***
	(10.591)	(11.038)	(10.394)	(10.359)
Total dividend dummy	-0.868***	-0.848***	-0.814***	-0.858***
	(-5.004)	(-4.884)	(-4.681)	(-4.937)
R&D expenditure	-0.008	-0.009	-0.009	-0.008
	(-1.028)	(-1.063)	(-1.120)	(-0.977)
Return on assets	-3.669***	-3.667***	-3.672***	-3.673***
	(-6.783)	(-6.775)	(-6.840)	(-6.788)
Constant	-0.080	-0.183	0.287	-0.086
	(-0.170)	(-0.389)	(0.630)	(-0.185)
Industry effect	Yes	Yes	Yes	Yes
Observations	3,042	3,042	3,042	3,042
R-squared	0.245	0.247	0.252	0.247

*** p<0.01

** p<0.05

* p<0.10 (Robust t-statistics in parentheses)

#### 4.4.2. COVID-related disclosure and employee turnover

Apparently, it is puzzling why firms will be making more COVID-related disclosures as it can significantly affect their financial performance. One important argument in favor of disclosure is that it is the company’s moral and legal obligation to reduce information asymmetry between the firm and shareholders and stakeholders [17]. Also, the extent and quality of corporate disclosure are reflections of better corporate governance and monitoring capability [88]. Additionally, firm employees—one of the most important stakeholders of a firm–may feel less confident about the financial stability and growth of the firm (that may lead to layoff) if the firm is not transparent during a turbulent period. This may lead to higher employee turnover.

Do firms benefit from more COVID disclosure in terms of employee retention? Do such firms make more efforts to retain employees during COVID-driven uncertain periods? To address these issues, we have examined the impact of COVID-related disclosure on employee turnover. The relation between COVID-related disclosure and employee turnover is shown in Table 12 (Models 1 –Model 4). We see that at the 1 percentile level, the coefficients for the *frequency of COVID-related disclosure* (-9.373), *contextual COVID-related positive word frequency* (-19.027), *contextual COVID-related negative word frequency* (-12.292), and *contextual COVID-related financial constraints word frequency* (-18.701) are all statistically significant. It lends credence to previous research showing that transparent disclosure establishes confidence among employees that lowers the rate of employee turnover [89]. Also, it may suggest that firms with better informational transparency make more efforts to retain their employees during uncertain periods.

**Table 12:** Relationship between COVID-related disclosure and employee turnover.

	DV: Employee turnover			
Variables	Model (1)	Model (2)	Model (3)	Model (4)
Frequency of COVID-related disclosure	-9.373***			
	(-4.190)			
Contextual COVID-related positive word frequency		-19.027***		
		(-4.686)		
Contextual COVID-related negative word frequency			-12.292***	
			(-8.676)	
Contextual COVID-related financial constraints word frequency				-18.701***
				(-5.230)
Herfindahl index	0.505	0.479	0.405	0.525
	(1.341)	(1.281)	(1.029)	(1.414)
Long-term debt to assets	-0.132***	-0.133***	-0.127***	-0.129***
	(-5.382)	(-5.403)	(-5.247)	(-5.315)
Firm size	0.027***	0.028***	0.026***	0.026***
	(9.902)	(10.243)	(9.566)	(9.594)
Total dividend dummy	-0.082***	-0.080***	-0.075***	-0.082***
	(-6.181)	(-6.024)	(-5.760)	(-6.132)
R&D expenditure	0.001	0.001	0.000	0.001
	(0.564)	(0.545)	(0.502)	(0.631)
Return on assets	0.023	0.023	0.022	0.022
	(1.517)	(1.539)	(1.494)	(1.463)
Constant	-0.190**	-0.205***	-0.134*	-0.201***
	(-2.424)	(-2.650)	(-1.659)	(-2.617)
Industry effect	Yes	Yes	Yes	Yes
Observations	3,005	3,005	3,005	3,005
R-squared	0.110	0.111	0.124	0.111

*** p<0.01

** p<0.05

* p<0.10 (Robust t-statistics in parentheses)

## 5. Conclusion

During the pandemic era, to understand the uncertainties and challenges associated with a firm’s operation, COVID-related disclosure has become quite critical for shareholders and other market participants [19]. However, for firm management, COVID-related disclosure is like a ‘double-edged sword’. On one hand, firms are supposed to report extensively on COVID-related developments and challenges in order to ensure a transparent disclosure environment with various stakeholders [9]. This will enhance trust with the stakeholders and alleviate litigation risks [90]. On the other hand, more COVID-related disclosure is likely to give a negative impression to the investors and stakeholders, which in turn might affect its operating and stock performance. Therefore, it is quite interesting and important to examine how firms manage their COVID-related disclosures. One of the major challenges in such an investigation is, there is no reliable or comprehensive measure to ascertain the level of COVID-related disclosure by a firm.

To address this gap, the main focus of this study has been to develop and validate various COVID-related dictionaries. More specifically, using a sample of publicly listed U.S. firms for the financial year of 2020, we have developed two types of COVID dictionaries (or COVID-related disclosure measurement tools): (a) overall COVID-dictionary (count of all COVID-related words/phrases) and (b) contextual COVID-dictionary (count of COVID related words/phrases preceded or followed by positive, negative, or financial constraints word). Subsequently, we have validated both types of COVID dictionaries by investigating their association with corporate liquidity events (e.g., dividend payment, dividend change). Following Bodnaruk, Loughran [10], we confirm that the overall COVID dictionary effectively predicts a firm’s liquidity event. We find similar results for contextual COVID dictionaries with a negative spin (i.e., COVID disclosures with a negative tone or an indication of financial constraints).

Our results further show that better-governed firms (e.g., greater board independence, and more female directors) tend to have more COVID-related disclosures, despite the fact that more COVID-related disclosures suppress a firm’s market-based stock performance (e.g. Tobin’s Q). Our results suggest that better-governed firms prefer greater transparency, even if it might hurt their market performance in the short run.

By developing effective dictionaries on COVID-related disclosures, this study paves the path for a better understanding of a firm’s COVID-related challenges and communication strategies. To the best of our knowledge, this is the first comprehensive study in this domain in the context of the U.S. market. We believe that the findings of this study will be quite useful for managers, regulators, shareholders, and other business partners. The regulators, for instance, could inspire the establishment of COVID-related disclosure guidelines. This will help make COVID-related reporting more consistent. Creditors and investors alike will be able to evaluate COVID-related challenges faced by a firm more consistently. Further, our findings will help firm management in setting up their COVID-related disclosure strategies.

### 5.1. Limitations of the study and future work

While we have developed and validated a systematic COVID-related disclosure dictionary, there are some limitations of the study. First, we have relied on 10-K documents (i.e. annual reports) to develop our dictionary. However, we recognize that there are multiple channels (e.g. earnings calls) for corporate disclosure. This may modify the dictionary to a certain extent. Second, we have relied on one-year corporate disclosure in this study. Over the years, the nature of COVID-related disclosures may evolve and new terminologies could be used. Third, in this study, we have used a semi-supervised NLP methodology, in which initial seed words and expanded wordlist selection may depend on researchers’ perceptions. This may induce some bias in dictionary building. Fourth, COVID-related disclosures may depend on other factors as well (such as managerial characteristics, firm’s risk-taking behavior). This may affect the validation of the COVID-related dictionary, as presented in this study. These issues might be addressed by future studies.

## Supporting information

S1 Table COVID dictionary building. (DOCX)

S2 Table Dictionary building steps summary. (DOCX)

S1 File Examples of WINDOW algorithm. (DOCX)

S2 File Data file. (XLSX)
