# Supplementary material for: Using 10-K text to gauge COVID-related corporate disclosure
Source: PLoS One. 2023 Mar 22;18(3):e0283138. doi: 10.1371/journal.pone.0283138 (PMC10032508; doi:10.1371/journal.pone.0283138)
Supplement: S1 Table — (DOCX) [file pone.0283138.s001.docx]

## S1 – COVID dictionary building

*S1 provides insight into the process of COVID dictionary creation. It contains detailed information of the steps involved in the dictionary development process such as: consideration of seed words, examination of the relevance of seed words, examination of the synonyms of words, proposition of additional COVID related words and their sources, and the presentation of final COVID dictionary.*

**Stage 1:** Considered LM COVID wordlist as initial seed list

Firstly, we considered the LM COVID wordlist (see Panel 1A) and generated synonyms of each of its word (see Panel 1C) by using the Word2vec model which was trained on MDA 2020 textual content. How we implemented the Word2vec model is explained in Section 3. Effectively, Word2vec generates a numerical representation of a word, which is generated based on what the textual content model is being trained on. It emerges that only 6 words from the LM Covid wordlist are present in MDA 2020 text (see Panel 1B). Further, we examined the synonyms of these 6 words and only considered relevant words (see Panel 1D for included and excluded words from the LM wordlist) for subsequent steps. Following are the LM wordlist and synonyms generated by the Word2vec model:

**Panel 1A:** LM Covid wordlist.

| LM Covid wordlist | 'pandemic'  'epidemic'  'corona_virus'  'contagious_disease'  'contagious_illness'  'infectious_disease'  'infectious_outbreak'  'fear_of_contagion'  'influenza_virus'  'avian_flu'  'h1n1'  'swine_flu'  'mers'  'ebola'  'localized_illnesses'  'health_concerns'  'outbreak_disease'  'outbreak_of_disease' |
| --- | --- |

**Panel 1B:** LM covid wordlist words present in MDA 2020 corpus.

| Included LM words | 'pandemic'  'epidemic'  'contagious_disease'  'infectious_disease'  'mers'  'ebola' |
| --- | --- |

**Panel 1C:** Internal validation of COVID-related LM words by examining their synonyms.

| **pandemic** | ['crisis', 'containment_mitigation', 'reoccurrence', 'coronavirus_disease_covid', 'covid', 'outbreak_novel_coronavirus', 'rapid_spread', 'novel_strain_coronavirus', 'spread_virus', 'continues_rapidly_evolve', 'try_contain', 'spread_throughout_world', 'virus', 'reemergence', 'not_contained', 'curb_spread', 'coronavirus_disease', 'recommended_containment_mitigation', 'variant_strains', 'continuously_evolving', 'rapidly_evolving_situation', 'coivd', 'developments_highly_uncertain', 'novel_coronavirus_disease', 'coronavirus_outbreak', 'aggravated', 'disrupted_global', 'dissipate', 'outbreaks', 'rapidly_evolve', 'vaccination_efforts', 'stalled', 'outbreak_novel', 'lock_downs', 'health_crises', 'remains_fluid', 'unprecedented', 'declared_national_emergency', 'declared_outbreak_novel_coronavirus', 'contagious_disease', 'scope_severity_duration', 'health_epidemics', 'transmissible', 'disruptions', 'disruption_caused', 'adversity', 'stay_home_orders', 'continues_unfold', 'fears', 'lockdown', 'resurgence_virus', 'turmoil', 'epidemic', 'widespread', 'dangers', 'persisting', 'length_severity'] |
| --- | --- |
| **epidemic** | 'health_epidemics', 'contagious_diseases', 'health_crises', 'pandemics_epidemics', 'public_health_crises', 'covid', 'epidemics', 'epidemics_pandemics', 'developments_highly_uncertain', 'concerning_severity', 'variant_strains', 'reoccurrence', 'reemergence', 'health_emergencies', 'strains_virus', 'pandemics', 'contagious', 'outbreaks', 'virulent', 'transmissible', 'outbreak_novel_coronavirus', 'emerge_concerning', 'continuously_evolving', 'contagion', 'calamities', 'harms', 'novel_coronavirus', 'numerous_evolving', 'actions_taken_contain', 'scope_severity', 'variants_virus', 'actions_taken_contain_treat', 'emerge_concerning_severity', 'precludes_prediction', 'recommended_containment_mitigation', 'scope_severity_duration', 'severe_acute', 'not_predicted_confidence', 'diplomatic', 'responses_thereto', 'outbreak', 'infectious_diseases', 'try_contain', 'far_reaching', 'contain_treat', 'hostilities', 'devastating', 'zika', 'taken_contain', 'health_crisis', 'resurgences', 'not_able_accurately_predict', 'terrorist_acts', 'societal', 'climatic', 'aggravated', 'public_health_crisis', 'coronavirus_sars', 'coronavirus_disease', 'resurgent', 'effectiveness_vaccines', 'unknowns', 'wuhan', 'declared_outbreak_novel_coronavirus', 'communicable', 'novel_strain', 'political_unrest', 'vaccination_efforts', 'containment_mitigation', 'outbreak_novel', 'uncertain_unpredictable', 'exacerbation', 'infectious', 'threatens', 'inoculations', 'turbulent', 'rapidly_evolving_situation', 'heightening', 'dangers', 'mutate', 'not_accurately_predicted', 'not_certainty', 'coronavirus_outbreak', 'disrupted_global', 'continues_rapidly_evolve', 'actions_taken_governmental_authorities', 'outbreak_novel_strain_coronavirus', 'sars_cov_virus'] |
| **contagious_disease** | 'contagious_diseases', 'health_crises', 'health_epidemics', 'transmissible', 'public_health_crises', 'pandemics_epidemics', 'resurgent', 'reoccurrence', 'try_contain', 'health_emergencies', 'epidemics', 'reemergence', 'calamities', 'developments_highly_uncertain', 'diplomatic', 'war_terrorism', 'variant_strains', 'severe_acute', 'covid', 'contagious', 'wuhan', 'declared_outbreak_novel_coronavirus', 'outbreak_novel_coronavirus', 'zika', 'outbreak_novel', 'taken_contain', 'political_unrest', 'virulent', 'not_contained', 'violence', 'harms', 'hostilities', 'containment_mitigation', 'instituting_quarantines', 'outbreaks', 'acts_war', 'vaccination_efforts', 'threatens', 'novel_strain', 'quarantines_travel', 'coronavirus_sars', 'recommended_containment_mitigation', 'contagion', 'proliferate', 'scope_severity', 'aggravated', 'economy_disrupted', 'coronavirus_disease', 'turbulent', 'inoculations', 'lock_downs', 'infectious', 'concerning_severity', 'measures_try', 'phenomena', 'coping', 'pathogenic', 'protectionist', 'stall', 'recession_depression', 'repercussions', 'outbreak_novel_strain_coronavirus', 'tensions', 'quarantines_stay', 'continuously_evolving', 'trade_tensions', 'confront', 'dangers', 'terrorist_acts', 'far_reaching', 'numerous_evolving', 'coivd', 'tropical_storms', 'actions_taken_governmental_authorities', 'scope_severity_duration', 'exacerbation', 'spread_throughout_world', 'communicable', 'straining', 'fears', 'continues_rapidly_evolve', 'place_stay', 'resurgences', 'reinstituting', 'rapid_spread', 'nongovernmental', 'outbreak', 'mandated_shutdowns', 'deadly', 'acute_respiratory', 'strain_coronavirus', 'strains_virus', 'vaccine_rollout', 'declared_national_emergency', 'vaccinate', 'novel_coronavirus_disease', 'infectious_diseases', 'health_emergency'] |
| **infectious_disease** | 'infectious_diseases', 'malaria', 'zika', 'coronavirus', 'severe_acute', 'hepatitis_virus', 'dengue', 'hmpv', 'infectious', 'transmissible', 'liver_diseases', 'deadly', 'cmv', 'sars_cov_virus', 'syncytial', 'microbiology', 'coronavirus_sars', 'multidrug', 'autoimmunity', 'cytomegalovirus', 'virulent', 'aeruginosa', 'oan', 'feline', 'genetic_diseases', 'exacerbations', 'acute_respiratory', 'fibrotic', 'sexually', 'pathogenic', 'hepatitis', 'microbial', 'pneumococcal', 'pulmonologists', 'nephrology', 'contagious_diseases', 'pulmonology', 'neurological', 'gastro', 'globulin', 'viral_infections', 'hsv', 'foodborne', 'neuromuscular', 'autoimmune', 'eradicating', 'modulates', 'autism', 'palliation', 'bicycle', 'amurin', 'eradication', 'cardiometabolic', 'neuroscience', 'mutate', 'avian', 'intracellular', 'inactivated', 'biofilm', 'enveloped', 'anti_viral', 'coping', 'parvovirus', 'chronic_hepatitis', 'vaccine_candidates', 'bcda', 'pseudomonas', 'glycosylation', 'oropharyngeal', 'lca', 'pathogens', 'addiction', 'afflicted', 'herpes', 'oxidative', 'autoimmune_diseases', 'palliative', 'infective', 'weeds', 'atopic', 'brii', 'ftd', 'autoimmune_disease', 'inflammatory_diseases', 'occlusive', 'cassette', 'allergic', 'microbes', 'cutis', 'immunology', 'radioisotopes', 'diagnosing', 'crohn', 'biodefense', 'gonorrhea', 'viruses', 'cov', 'vaso', 'shingles', 'combatting', 'contagious', 'reoccurrence', 'reproductive', 'bacterial_infections', 'metabolic_diseases', 'monogenic', 'mnpr', 'life_threatening', 'antimicrobial', 'humoral', 'fusion_protein', 'deleterious', 'novel_strain', 'rare_disease', 'eradicate', 'cgrp', 'papillomavirus', 'hyperkalemia', 'frontotemporal', 'exacerbation', 'exosome', 'savadx', 'mgd', 'adenovirus', 'mmts', 'contagiosum', 'immuno_oncology', 'urological', 'dlas', 'degeneration', 'allergies', 'respiratory', 'oncologic', 'diagnose', 'lethality', 'obstruction', 'nucleotides', 'tats', 'phage', 'modulation', 'immunogenic', 'gpcrs', 'evlp', 'tnx', 'neurologic', 'communicable', 'angioedema', 'sars_cov', 'oncological', 'dysregulation', 'rare_genetic', 'novel_therapeutics', 'health_crises', 'illnesses', 'fight_against', 'anxiety', 'high_unmet', 'manocept', 'mdps', 'copd', 'monovalent', 'rare_diseases', 'pneumonitis', 'conjunctivitis', 'inhaled', 'pathogen', 'germs', 'pancreatitis', 'implicated', 'dismutase', 'intractable'] |
| **mers** | ['cytology', 'hsv', 'kills', 'evlp', 'worms', 'neural', 'teeth', 'diary', 'weapon', 'endoscopes', 'lymphedema', 'passages', 'monovalent', 'syncytial', 'herpes', 'scars', 'oropharyngeal', 'disable', 'radial', 'cytomegalovirus', 'tricuspid', 'lamp', 'swabs', 'sexually', 'adjuvants', 'uploads', 'encrypted', 'endoluminal', 'touchscreen', 'electroporation', 'disabling', 'adenovirus', 'avian'] |
| **ebola** | ['zika', 'syncytial', 'monovalent', 'cytomegalovirus', 'avian', 'herpes', 'aeruginosa', 'pseudomonas', 'deadly', 'cmv', 'organisms', 'frontotemporal', 'vulvar', 'multiforme', 'hepatitis_virus', 'malaria', 'severe_acute', 'hsv', 'dengue', 'esophagitis', 'sexually', 'hyperkalemia', 'multidrug', 'adjuvanted', 'cell_transplant'] |

**Panel 1D:** Included and Excluded LM COVID wordlist.

| **LM COVID words** | |
| --- | --- |
| **Included** | **Excluded** |
| 'pandemic' | 'mers' |
| 'epidemic' | 'ebola' |
| 'contagious_disease' |  |
| 'infectious_disease' |  |

**Stage 2:** **Finding additional COVID-related words**

After carefully analyzing synonyms of LM wordlist words, we came up with additional potential COVID-related words (see Panel 2A). To test whether they are relevant to COVID-related words, we validated these additional words by examining their synonyms (see Panel 2B).

**Panel 2A:** Additional COVID-related words and their source.

| **Additional Covid related words (potential)** | **Identified from synonyms of words** | **From corpus and combination of COVID related words** |
| --- | --- | --- |
| coronavirus | infectious_disease | **−** |
| coronavirus_pandemic | **−** | From corpus |
| covid_pandemic | **−** | From corpus |
| covid | pandemic, epidemic, contagious_disease | **−** |
| novel_coronavirus | epidemic | **−** |
| novel_strain_coronavirus | pandemic | **−** |
| covid_outbreak | **−** | From corpus |
| resurgence_covid | **−** | From corpus |
| health_crisis | epidemic | **−** |
| public_health_crisis | epidemic | **−** |
| outbreak | epidemic, contagious_disease | **−** |
| novel_strain | epidemic, contagious_disease, infectious_disease | **−** |
| novel_coronavirus_disease | pandemic, contagious_disease | **−** |
| coronavirus_disease | pandemic, epidemic, contagious_disease, infectious_disease | **−** |

**Panel 2B:** Internal validation of additional COVID-related words by examining their synonyms.

| **coronavirus** | ['coronavirus_disease_covid', 'coronavirus_sars', 'transmissible', 'covid', 'outbreak_novel_coronavirus', 'variant_strains', 'novel_coronavirus_disease', 'coronavirus_disease', 'severe_acute', 'contagious', 'reoccurrence', 'outbreak_novel_strain_coronavirus', 'variants_virus', 'virus', 'sars_cov_virus', 'outbreak_novel', 'variants', 'reemergence', 'contagious_diseases', 'declared_outbreak_novel_coronavirus', 'declared_national_emergency', 'rapid_spread', 'declared_outbreak', 'emerge_concerning', 'strains_virus', 'covid', 'actions_taken_governmental_authorities', 'inoculations', 'strain_coronavirus', 'coronavirus_outbreak', 'health_emergencies', 'deadly', 'curb_spread', 'continuously_evolving', 'contagious_disease', 'health_emergency', 'wuhan'] |
| --- | --- |
| **coronavirus_pandemic** | ['outbreak_novel_coronavirus', 'uncertainly', 'rapid_spread', 'disruption_caused', 'covid', 'repercussions', 'coronavirus_disease', 'coronavirus_disease_covid', 'continues_rapidly_evolve', 'health_epidemics', 'situation_surrounding', 'contagious_disease', 'disrupted_global', 'given_dynamic_nature', 'outbreak_novel', 'pauses', 'rapidly_evolving_situation', 'curb_spread', 'wuhan', 'disruption', 'crisis', 'transmissible', 'scope_severity', 'adversity', 'coronavirus_outbreak', 'epidemic', 'health_crises', 'variant_strains', 'resurgent', 'unprecedented', 'novel_coronavirus_disease', 'recession_depression', 'outbreak_novel_strain_coronavirus'] |
| **covid_pandemic** | ['coronavirus_disease_covid', 'disrupted_global', 'disruption_caused', 'pauses', 'coronavirus_outbreak', 'covid', 'coronavirus_disease', 'continues_rapidly_evolve', 'aggravated', 'containment_mitigation', 'taken_contain', 'disruptions_caused', 'repercussions', 'rapidly_evolving_situation', 'stalled', 'crisis', 'impacts', 'unprecedented', 'worsening_global', 'reoccurrence', 'far_reaching', 'outbreak_novel_coronavirus', 'economic_slowdown', 'curb_spread', 'recession_depression', 'given_dynamic_nature', 'health_crises', 'economic_downturn', 'situation_surrounding', 'global_economy', 'disruptions', 'remains_fluid', 'continuously_evolving', 'developments_highly_uncertain', 'elective_surgical', 'rapid_spread', 'severely_impacted', 'covd', 'actively_monitor_situation', 'uncertainly', 'lock_downs', 'scope_severity', 'mandated_shutdowns', 'try_contain', 'remain_cautious', 'cutbacks', 'dynamic_nature', 'navigates', 'contagious_diseases', 'wuhan', 'certainly', 'turmoil', 'numerous_evolving', 'outbreak_novel', 'responses_thereto', 'unabated', 'global_economy_disrupted_global', 'disruption', 'global_economic_slowdown', 'challenges_posed', 'disrupted_global_supply_chains', 'persisting', 'visitations', 'dissipated', 'lingering', 'contagious_disease', 'abruptly', 'reemergence', 'evolving_situation', 'onset_pandemic', 'precautionary_measures', 'spread_throughout_world', 'economic_fallout', 'not_materially_adversely', 'precludes_prediction', 'steps_protect', 'spread_virus', 'mitigate_spread_virus', 'turbulent', 'outbreaks', 'dissipate', 'slowdowns_shutdowns', |
| **covid** | ['coronavirus_disease_covid', 'virus', 'covd', 'coronavirus_disease', 'reoccurrence', 'containment_mitigation', 'coivd', 'taken_contain', 'lock_downs', 'elective_surgical', 'try_contain', 'variant_strains', 'outbreak_novel', 'wuhan', 'coronavirus_sars', 'pauses', 'novel_coronavirus_disease', 'coronavirus_outbreak', 'inoculations', 'effectiveness_vaccines', 'disrupted_global', 'outbreak_novel_strain_coronavirus', 'rapid_spread', 'contagious_diseases', 'outbreak_novel_coronavirus', 'severe_acute', 'health_crises', 'curb_spread', 'dissipated', 'developments_highly_uncertain', 'reemergence', 'mitigate_spread_virus', 'combat_virus', 'reinstituting', 'loosening', 'mandated_shutdowns', 'resurgences', 'health_epidemics', 'advisories', 'stall', 'strains_virus', 'continuously_evolving', 'not_contained', 'soften', 'resurgent', 'stalled', 'spread_virus', 'continues_rapidly_evolve', 'logistical_challenges', 'transmissible', 'vaccination_efforts', 'spread_throughout_world', 'recommended_containment_mitigation', 'contagious_disease', 'reinstitution', 'outbreaks', 'contagious', 'health_emergencies', 'dangers', 'virulent', 'contain_virus', 'disruption_caused', 'aggravated', 'fears', 'dissipate', 'visitations', 'unfold', 'variants_virus', 'declared_outbreak_novel_coronavirus', 'resurgence_virus', 'sars_cov_virus', 'reimposition', 'quarantines_travel', 'rapidly_evolve'] |
| **novel_coronavirus** | ['outbreak_novel_strain_coronavirus', 'coronavirus_disease', 'outbreak_novel_coronavirus', 'coronavirus_disease_covid', 'novel_coronavirus_disease', 'coronavirus_sars', 'outbreak_novel', 'declared_outbreak', 'declared_national_emergency', 'spread_throughout_world', 'declared_outbreak_novel_coronavirus', 'severe_acute', 'covid', 'recommended_containment_mitigation', 'transmissible', 'president_declared', 'strain_coronavirus', 'contagious_disease', 'rapid_spread', 'contagious_diseases', 'sars_cov_virus', 'health_epidemics', 'continues_rapidly_evolve', 'surfaced', 'wuhan', 'epidemic', 'variant_strains', 'cov', 'health_emergencies', 'reoccurrence', 'health_crises', 'reemergence', 'coronavirus_outbreak', 'health_emergency', 'taken_contain', 'contagious', 'virulent', 'containment_mitigation', 'public_health_crises', 'pandemics_epidemics', 'coivd', 'disrupted_global', 'try_contain', 'epidemics_pandemics', 'epidemics', 'global_economy_disrupted_global', 'situation_surrounding', 'implementing_numerous', 'outbreaks', 'continuously_evolving', 'responses_thereto', 'zika', 'developments_highly_uncertain', 'pathogenic', 'challenges_posed', 'repercussions', 'dangers', 'infectious', 'precludes_prediction', 'concerning_severity', 'prediction_ultimate', 'navigates', 'devastating', 'resurgent', 'actions_taken_governmental_authorities', 'threatens', 'not_contained', 'curb_spread', 'scope_severity', 'prevent_spread', 'far_reaching', 'actions_taken_contain_treat', 'emerge_concerning', 'mutate', 'governmental_responses', 'coronaviruses', 'profound', 'resurgences', 'virus', 'actively_monitor_situation', 'measures_try', 'rapidly_evolving_situation', 'inoculations', 'effectiveness_vaccines', 'intended_prevent', 'exacerbation', 'stall', 'decisively', 'scope_severity_duration', 'public_health_emergency', 'turmoil', 'proclamation', 'coping', 'infectious_diseases', 'deadly', 'disrupted_global_supply_chains', 'proliferate', 'crisis', 'actions_taken_contain', |
| **novel_strain_coronavirus** | ['outbreak_novel_strain_coronavirus', 'coronavirus_sars', 'novel_coronavirus_disease', 'declared_outbreak', 'outbreak_novel', 'outbreak_novel_coronavirus', 'coronavirus_disease_covid', 'surfaced', 'spread_throughout_world', 'declared_outbreak_novel_coronavirus', 'strain_coronavirus', 'coronavirus_disease', 'severe_acute', 'president_declared', 'recommended_containment_mitigation', 'declared_national_emergency', 'covid', 'transmissible', 'rapid_spread', 'continues_rapidly_evolve', 'taken_contain', 'pathogenic', 'contagious', 'contagious_disease', 'virulent', 'health_emergency', 'containment_mitigation', 'sars_cov_virus', 'try_contain', 'stall', 'wuhan', 'reoccurrence', 'situation_surrounding', 'deadly', 'contagious_diseases', 'implementing_numerous', 'detected', 'reemergence', 'variant_strains', 'global_economy_disrupted_global', 'disrupted_global', 'syncytial', 'zika', 'public_health_emergency', 'health_emergencies', 'health_epidemics', 'resurgent', 'hmpv', 'infectious', 'coronavirus_outbreak', 'coronaviruses', 'not_contained', 'national_emergency', 'avian', 'health_crises', 'evolving_situation', 'dangers', 'viral_infections', 'profound', 'oan', 'threatens', 'dengue', 'measures_try', 'public_health_crises', 'multidrug', 'taskforce', 'afflicted', 'pandemics_epidemics', 'complication', 'restrain', 'repercussions', 'inactivated', 'vaccination_efforts', 'pneumonia', 'germs', 'reinstituting', 'mutate', 'devastating', 'harms', 'combating', 'decisively', 'coping', 'proliferate', 'monovalent', 'proclamation', 'combat_virus', 'ebola', 'exacerbation', 'ameliorate', 'exacerbations', 'reimposition', 'fever', |
| **covid_outbreak** | ['coronavirus_disease_covid', 'curb_spread', 'outbreak_novel_coronavirus', 'coronavirus_disease', 'spread_throughout_world', 'developments_highly_uncertain', 'reemergence', 'coronavirus_outbreak', 'continues_rapidly_evolve', 'contagious_disease', 'reoccurrence', 'disrupted_global', 'health_emergency', 'declared_national_emergency', 'rapid_spread', 'recommended_containment_mitigation', 'containment_mitigation', 'try_contain', 'health_emergencies', 'outbreak_novel', 'remains_fluid', 'outbreak_novel_strain_coronavirus', 'novel_coronavirus_disease', 'continuously_evolving', 'contagious_diseases', 'scope_severity', 'health_crises', 'president_declared', 'coronavirus_sars', 'wuhan', 'rapidly_evolving_situation', 'not_contained', 'declared_outbreak_novel_coronavirus', 'concerning_severity', 'precludes_prediction', 'repercussions', 'severity_duration', 'economic_fallout', 'health_epidemics', 'given_dynamic_nature', 'evolving_situation', 'actions_taken_governmental_authorities', 'declared_outbreak', 'contagious', 'uncertainly', 'not_certainty', 'emerge_concerning', 'scope_severity_duration', 'virus', 'prediction_ultimate', 'uncertain_unpredictable', 'transmissible', 'governmental_responses', 'epidemic', 'recession_depression', 'slowdowns_shutdowns', 'resurgence', 'strain_coronavirus', 'severe_acute', 'strains_virus', 'resurgences', 'broader_implications', 'global_economies', 'pandemics', 'aggravated', 'depend_numerous_evolving', 'public_health_emergency', 'abruptly', 'quarantines_stay', 'pauses', 'severely_impacted', 'mandated_shutdowns', 'economy_disrupted', 'remain_cautious', 'dynamic_nature'] |
| **resurgence_covid** | ['resurgences', 'resurgence', 'surges', 'outbreaks', 'resurgence_virus', 'variants_virus', 'lockdowns', 'reimposition', 'containment_mitigation', 'variant_strains', 'strains_virus', 'loosening', 'spikes', 'infections', 'waves', 'reimplemented', 'reinstitution', 'lock_downs', 'stalled', 'virus', 'contagion', 'contagious', 'reinstituting', 'rapid_spread', 'reemergence', 'stall', 'inoculations', 'reoccurrence', 'vaccination_efforts', 'reintroduced', 'reopenings', 'vaccine_rollout', 'dissipate', 'resurgent', 'mitigate_spread_virus', 'contagious_disease', 'taken_contain', 'unfortunately', 'reinstitute', 'spread_throughout_world', 'try_contain', 'combat_virus', 'setbacks', 'continuously_evolving', 'spread_virus', 'reimpose', 'quarantines_shelter_place_orders', 'not_contained', 'declared_outbreak_novel_coronavirus', 'stay_home_orders', 'quarantines_stay', 'rollouts', 'health_crises', 'aggravated', 'developments_highly_uncertain', 'coronavirus_outbreak', 'variants', 'recurrences', 'concerning_severity', 'quarantines_travel', 'pace_recovery', 'transmissible', 'mandated_shutdowns', 'abating', 'effectiveness_vaccines', 'populous', 'health_emergencies', 'impositions', 'abruptly', 'elective_surgical_procedures', 'worsens', 'surging', 'coronavirus_sars', 'travel_bans'] |
| **health_crisis** | ['outbreak_novel_coronavirus', 'responses_thereto', 'outbreak_novel', 'situation_surrounding', 'continues_rapidly_evolve', 'health_crises', 'health_epidemics', 'reoccurrence', 'disrupted_global', 'taken_contain', 'not_contained', 'global_economies', 'coronavirus_disease_covid', 'coronavirus_outbreak', 'navigates', 'coronavirus_disease', 'coping', 'challenges_posed', 'scope_severity', 'contagious_diseases', 'declared_outbreak_novel_coronavirus', 'containment_mitigation', 'aggravated', 'recommended_containment_mitigation', 'far_reaching', 'spread_throughout_world', 'repercussions', 'remain_cautious', 'continuously_evolving', 'widespread', 'reemergence', 'contagious_disease', 'outbreak_novel_strain_coronavirus', 'variant_strains', 'global_economic_slowdown', 'remains_fluid', 'broader_implications', 'epidemic', 'recession_depression', 'economic_fallout', 'dissipate', 'decisively', 'economies', 'turmoil', 'resurgent', 'health_emergencies', 'rapidly_evolving_situation', 'inoculations', 'rapidly_evolve', 'rapid_spread', 'scope_severity_duration', 'developments_highly_uncertain', 'dangers', 'worsening_global', 'adversity', 'epidemics', 'transmissible', 'public_health_crises', 'turbulent', 'declared_national_emergency', 'turbulence', 'unfold', 'actions_taken_governmental_authorities', 'threatens', 'aftermath', 'continues_unfold', 'disruptions_caused', 'logistical_challenges', 'novel_coronavirus_disease', 'numerous_evolving', 'outbreaks', 'economic_downturn', 'microeconomic', 'wuhan', 'health_emergency'] |
| **public_health_crisis** | ['taken_contain', 'containment_mitigation', 'reoccurrence', 'rapid_spread', 'continues_rapidly_evolve', 'quarantines_stay', 'repercussions', 'try_contain', 'spread_throughout_world', 'outbreak_novel_coronavirus', 'implementing_numerous', 'instituting_quarantines', 'reemergence', 'contagious_disease', 'health_epidemics', 'mitigate_spread_virus', 'measures_try', 'outbreak_novel', 'health_emergencies', 'responses_thereto', 'wuhan', 'contagious_diseases', 'coronavirus_outbreak', 'coronavirus_disease', 'recommended_containment_mitigation', 'declared_outbreak_novel_coronavirus', 'mandated_closures', 'developments_highly_uncertain', 'disrupted_global_supply_chains', 'epidemic', 'mandated_shutdowns', 'economy_disrupted', 'transmissible', 'health_crises', 'continuously_evolving', 'reacted', 'abruptly', 'virulent', 'global_economy_disrupted_global', 'variant_strains', 'declared_national_emergency', 'scope_severity', 'coronavirus_disease_covid', 'prevent_spread', 'novel_coronavirus_disease', 'contain_virus', 'disruption', 'health_emergency', 'contagious', 'far_reaching', 'adaptations', 'rapidly_evolving_situation', 'challenges_posed', 'coronavirus_sars', 'resurgent', 'concerning_severity', 'disrupted_global', 'quarantines_travel_restrictions', 'numerous_evolving', 'dangers', 'public_health', 'public_health_crises', 'quarantines_travel', 'travel_bans_restrictions_quarantines', 'actively_monitor_situation', 'not_contained', 'epidemics', 'president_declared', 'reimpose', 'travel_bans_restrictions', 'fears', 'governmental_responses', 'stay_home_orders', 'governments_around_world', 'border_closures', 'shelter_place_orders', 'quarantines_shelter_place_orders', 'slow_spread', 'proclamations', 'contain_treat', 'situation_surrounding', 'vaccination_efforts', 'outbreak_novel_strain_coronavirus', 'precludes_prediction', 'turmoil', 'proclamation', 'effectiveness_vaccines' , 'quarantines_shelter', 'declared_outbreak', 'vaccine_rollout'] |
| **outbreak** | ['outbreak_novel_coronavirus', 'taken_contain', 'reoccurrence', 'variant_strains', 'reemergence', 'concerning_severity', 'outbreaks', 'contagious_disease', 'virus', 'spread_throughout_world', 'spread_virus', 'rapid_spread', 'actions_taken_governmental_authorities', 'contagious', 'contagious_diseases', 'health_emergencies', 'containment_mitigation', 'resurgence', 'developments_highly_uncertain', 'emerge_concerning', 'outbreak_novel_strain_coronavirus', 'resurgences', 'actions_taken_contain', 'health_epidemics', 'try_contain', 'vaccination_efforts', 'coronavirus_disease', 'combat_virus', 'transmissible', 'outbreak_novel', 'covid', 'recommended_containment_mitigation', 'contain_treat', 'coronavirus_disease_covid', 'emerge_concerning_severity', 'coronavirus_outbreak', 'continuously_evolving', 'declared_outbreak_novel_coronavirus', 'contagion', 'scope_severity_duration', 'variants_virus', 'scope_severity', 'declared_national_emergency', 'uncertain_unpredictable', 'epidemic', 'health_crises', 'contain_virus', 'continues_rapidly_evolve', 'novel_coronavirus_disease', 'severe_acute', 'duration_severity', 'virulent', 'not_contained', 'strains_virus', 'curb_spread', 'waves', 'disrupted_global', 'strain_coronavirus', 'health_emergency', 'declared_outbreak', 'preventative_protective', 'resurgence_virus', 'lock_downs', 'resurgent', 'severity_duration', 'responses_thereto', 'stall', 'global_economy', 'duration_scope', 'rapidly_evolving_situation', 'pandemics', 'situation_surrounding', 'wuhan', 'quarantines_travel', 'president_declared', 'governmental_responses', 'lockdowns', 'reinstituting', 'instituting_quarantines', 'quarantines_stay', 'strains', 'surfaced', 'epidemics_pandemics', 'epidemics', 'variants', 'coivd', 'quarantines_travel_restrictions', 'precludes_prediction', 'stay_home_orders', 'advent', 'reimposed', 'public_health_crises', 'recession_depression', 'proclamation', 'spread', 'taken_proactive', 'shut_downs', 'quarantines_shelter', 'preventative_measures', 'crisis', 'economic_downturn', 'abruptly', 'rapidly_evolve', 'length_severity', 'public_health', 'global_economic_slowdown', 'fears', 'broader_implications'] |
| **novel_strain** | ['coronavirus_sars', 'outbreak_novel_strain_coronavirus', 'severe_acute', 'mutate', 'novel_coronavirus_disease', 'declared_outbreak', 'outbreak_novel', 'coronavirus_disease', 'coronavirus_disease_covid', 'declared_outbreak_novel_coronavirus', 'transmissible', 'outbreak_novel_coronavirus', 'president_declared', 'spread_throughout_world', 'strain_coronavirus', 'recommended_containment_mitigation', 'declared_national_emergency', 'virulent', 'pathogenic', 'deadly', 'zika', 'contagious', 'rapid_spread', 'syncytial', 'wuhan', 'avian', 'complication', 'contagious_diseases', 'dengue', 'multidrug', 'contagious_disease', 'exacerbation', 'continues_rapidly_evolve', 'devastating', 'taken_contain', 'sars_cov_virus', 'acute_respiratory' , 'try_contain', 'reemergence', 'coronaviruses', 'afflicted', 'resurgent', 'degeneration', 'variant_strains', 'hmpv', 'hepatitis_virus', 'chronic_hepatitis', 'hyperkalemia', 'fever', 'elevations', 'viral_infections', 'monovalent', 'reoccurrence', 'vaso', 'aeruginosa', 'health_emergency', 'exacerbations', 'dangers', 'recessive', 'atopic', 'health_crises', 'containment_mitigation'] |

**Stage 3:** By merging the relevant words from the LM dictionary and additional COVID related words, we created the final COVID wordlist (see Panel 3A).

**Panel 3A:** Final Covid wordlist.

| Final Covid wordlist | pandemic  epidemic  contagious_disease  infectious_disease  coronavirus  coronavirus_pandemic  covid_pandemic  covid  novel_coronavirus  novel_strain_coronavirus  covid_outbreak  resurgence_covid  health_crisis  public_health_crisis  outbreak  novel_strain  novel_coronavirus_disease  coronavirus_disease |
| --- | --- |

Please note that, we have used Regex (a Python library) to eliminate “E-mails, URLs, punctuations, new line characters, single characters, digits (i.e., numbers), and extra spaces”. Which implies that searching for word ‘Covid’ itself will account for COVID-19 or COVID19 words.
