# Supplementary material for: Using 10-K text to gauge COVID-related corporate disclosure
Source: PLoS One. 2023 Mar 22;18(3):e0283138. doi: 10.1371/journal.pone.0283138 (PMC10032508; doi:10.1371/journal.pone.0283138)
Supplement: S2 Table — (DOCX) [file pone.0283138.s002.docx]

## S2 – Dictionary building steps summary

*S2 presents a summary of the steps followed in developing the COVID dictionary.*

|  |  |
| --- | --- |
| Step 1 | Considered LM COVID wordlist as initial seed word list (see S1. Panel 1A) |
| Step 2 | Obtained synonyms of the LM words which are present in the MDA 2020 corpus (see S1, Panel 1B, Panel 1C) |
| Step 3 | Validated the LM words using synonyms and filtered relevant words for subsequent steps (see S1, Panel 1B) |
| Step 4 | Identified additional COVID related words (see S1, Panel 2A) |
| Step 5 | Validated the additional COVID words by examining their synonyms (see S1, Panel 2B) |
| Step 6 | Combined words from the included/filtered list of LM words and additional COVID words to come up with final COVID dictionary (see S1, panel 3A) |
