# Supplementary material for: Using 10-K text to gauge COVID-related corporate disclosure
Source: PLoS One. 2023 Mar 22;18(3):e0283138. doi: 10.1371/journal.pone.0283138 (PMC10032508; doi:10.1371/journal.pone.0283138)
Supplement: S1 File — (DOCX) [file pone.0283138.s003.docx]

## S3 – Examples of Window Algorithm

*S3 includes examples taken from the MDA corpus, which serve to demonstrate the WINDOW algorithm. The highlighted purple words are from the COVID dictionary, while those in yellow are from the LM and BLM dictionaries, which consist of Positive words, Negative words, and Financial Constraints words.*

**Examples:**

1. During much of 2020, the novel coronavirus (SARS-CoV-2 or COVID-19), recognized as a pandemic by the World Health Organization, caused significant economic effects where we operate, including temporary closures of many businesses and reduced consumer spending due to shelter-in-place, stay-at-home and other governmental actions. Those orders and the uncertainty surrounding COVID-19 had broad financial market effects and caused significant market disruption and volatility.
2. The COVID-19 pandemic slowed the growth of our premium revenues for 2020, including new business written premiums. Premium growth by segment is discussed below in Financial Results. For future periods, renewal premium or new business premium amounts could further decline if the basis for policy premiums, such as sales and payrolls of businesses we insure, decrease as a result of the pandemic and a weakened economy. In addition, the ultimate effects of past or future government-ordered actions, including moratoriums or deferral of premium payments related to our insurance policies, are uncertain and may further adversely affect premium growth.
3. During 2020, pandemic-related incurred losses and expenses totaled $85 million. The total included $30 million for legal expenses in defense of business interruption claims, $19 million for Cincinnati Re® losses, $12 million for Cincinnati Global Underwriting Ltd.SM (Cincinnati Global) losses, $8 million for credit losses related to uncollectible premiums and $16 million for the Stay-at-Home policyholder credit for personal auto policies.
4. Loss experience for our insurance operations is influenced by many factors, and higher catastrophe losses were the main driver of the 2020 increase in losses and loss expenses, compared with 2019. Pandemic-related amounts totaled approximately $36 million and were discussed in more detail in Corporate Financial Highlights of Management’s Discussion and Analysis. For 2020, loss experience before catastrophe effects for our commercial lines insurance segment continued to improve. The main contributor to the improvement was the ratio for the accident year 2020 loss and loss expenses before catastrophe losses. The improvement was driven by our commercial casualty and commercial auto lines of business, while commercial property increased by 1.1 points and workers' compensation increased by 0.9 points. The unfavorable change for commercial property included 3.0 points for $30 million of legal expenses for the defense of business interruption claims, related to the pandemic, incurred during 2020. The unfavorable change for workers' compensation reflected average percentage price changes that have decreased in the mid-single-digit range in several recent quarters.
5. During 2020, the COVID-19 pandemic and related economic effects caused volatility in the fair values of securities discussed below in Total Investment Gains and Losses. Our fixed-maturity and equity portfolios experienced a decrease in valuation during the first quarter of 2020, in large part due to the volatility and economic uncertainty caused by the coronavirus outbreak that affected various sectors of our portfolio. During the first quarter of 2020, already low oil prices and the sudden demand drop in related products due to governmental actions, such as shelter-in-place orders, contributed to the energy sector accounting for most of the write-downs of impaired securities in the tables below. During the last three quarters of 2020, valuations increased for a significant portion of our fixed-maturity and equity portfolios.
6. The COVID-19 pandemic and related economic effects slowed the rate of our premium growth in 2020. Most states where we market our products issued mandates or requests such as moratoriums on policy cancellations or nonrenewals for nonpayments of premiums, forbearance on premium collections, waivers of late payment fees, and extended periods in which policyholders may make their missed payments. Extended or future moratoriums and deferral of premiums may disrupt cash flows while also increasing credit risk from policyholders struggling to make timely premium payments.
7. While we have experienced improvements in our business since the early stages of the COVID-19 pandemic, it has nonetheless negatively impacted us and our customers, counterparties, employees, and third-party service providers since the spread of the disease began to accelerate in March 2020. At this time, we cannot yet be confident in the extent of this negative impact or the trajectory of the macroeconomic outlook—especially with the most recent resurgence of COVID-19 in much of the United States and ongoing uncertainties around the efficacy, availability, acceptance, and distribution of vaccines and other medical treatments—and the adverse effects on our business, financial position, results of operations, and prospects could be significant.
8. The credit performance of our portfolio of loans has been impacted by the pandemic. Before the pandemic, the national unemployment rate was below 4% for an extended period and was projected to sustain those levels into the foreseeable future. Additionally, most widely available forecasts assumed that a moderate rate of growth would continue throughout 2020. The impact of the lockdowns, stay-in-place directives, and other restrictive actions issued and enforced by federal, state, and local officials as a result of the pandemic, contributed to a significant increase in the national unemployment rate, which rose to a peak of 14.7%, as adjusted, in April before initially moderating at a faster-than-expected-pace to 7.9%, as adjusted, as of September 30, 2020. In the fourth quarter of 2020, the national unemployment rate stabilized—particularly in November and December—ending at 6.7% as of December 31, 2020. Similarly, the rate of economic growth, as measured by GDP, declined sharply on a quarter-over-quarter seasonally adjusted annualized rate basis in the second quarter by 31.7%, and rebounded in the third and fourth quarters of 2020 as the economy grew by 33.1%, as adjusted, and 4.0%, respectively, quarter-over-quarter based on this measure. On a year-over-year basis, GDP contracted by 2.5%.
9. The deterioration and uncertainty in the macroeconomic environment experienced in 2020 from the pandemic and related mitigation efforts drove a significant increase in our allowance for loan losses and provision for credit losses. Our provision expense for credit losses increased $441 million to $1.4 billion for the year ended December 31, 2020, compared to 2019. While the macroeconomic environment rebounded significantly in the third quarter and stabilized in the fourth quarter of 2020, the increase in provision expense year-over-year reflects the significant deterioration observed since the inception of the pandemic. During the second half of March 2020, the U.S. economy experienced a significant deterioration driven by the COVID-19 pandemic, which impacted our allowance for loan losses. During the first quarter, we recorded an additional $602 million of provision expense for credit losses associated with the deterioration in the macroeconomic outlook from COVID-19. During the second quarter of 2020, we incurred a total provision expense of $287 million, which included $128 million attributable to the macroeconomic environment and other factors aside from changes in portfolio size and incremental net charge-offs. During the third quarter of 2020, we incurred a total provision expense of $147 million, which included a reduction in reserves of $34 million attributable to the improving macroeconomic environment—predominately the unemployment rate, and other factors aside from changes in portfolio size and incremental net charge-offs. During the fourth quarter of 2020, we recorded total provision expense of $102 million, which included a further reduction in reserves of $114 million attributable to the continued economic recovery, and other factors aside from changes in portfolio size and incremental net charge-offs. During the year ended December 31, 2020, the provision for credit losses was also impacted by the adoption of CECL, as further described in Note 1 to the Consolidated Financial Statements.
10. Public equity markets have experienced significant volatility as a result of the pandemic, which has impacted our investment securities portfolio. For the year ended December 31, 2020, we recognized net realized and unrealized gains on equity securities of $107 million and $29 million, respectively. Given the unpredictability of COVID-19 and its direct and indirect effects on market conditions, it is possible that unusual volatility in the equity markets could positively or negatively impact our results.
11. Total consumer automotive loan and operating lease originations decreased by $1.2 billion for the year ended December 31, 2020, compared to 2019. The decrease for the year ended December 31, 2020, compared to the prior year, was primarily due to the impacts of the COVID-19 pandemic, including the temporary shutdown or restriction of front-end operations of automotive dealers during the second quarter of 2020. These restrictions, along with the industry-wide halt of new vehicle production, drove a significant decrease in industry automotive light vehicle sales.
12. Our lending and finance receivable balances have been impacted by the COVID-19 pandemic. Through the second quarter of 2020, as governments acted to temporarily close or restrict the operations of businesses, including automotive dealers, and as many consumers and businesses changed their behavior in response to government mandates and advisories to sharply restrain commercial and social interactions, we experienced significant reductions in our consumer automotive loan applications.
13. Although the macroeconomic outlook improved modestly during the second half of 2020, the future direct and indirect impact of COVID-19 on our businesses, the results of operations and the financial condition of the Corporation remains highly uncertain. Should current economic conditions persist or deteriorate, this macroeconomic environment will have a continued adverse effect on our businesses and the results of operations and could have an adverse effect on our financial condition.
14. Net income was $17.9 billion or $1.87 per diluted share in 2020 compared to $27.4 billion or $2.75 per diluted share in 2019. The decline in net income was primarily due to higher provision for credit losses driven by the weaker economic outlook related to COVID-19 and lower net interest income.
15. The provision for credit losses increased from $1.9 billion to $5.4 billion primarily due to the weaker economic outlook related to COVID-19. Non-interest expenses increased $442 million to $7.4 billion primarily driven by investments in the business and incremental expenses to support customers and employees during the pandemic.
16. The COVID-19 pandemic has disrupted global financial markets and negatively affected supply and demand across a broad range of industries and has caused disruption to our customers, vendors, and employees. This pandemic has had a significant impact on our business, financial condition, and results of operations during the year, including the impairment of the goodwill previously allocated to our Retail Banking and Wealth Management and Private Banking reporting units and an increase to the provision for credit losses on our loan portfolio.
17. The circumstances around this pandemic will continue to impact our business in future periods. Should the current economic conditions persist or deteriorate further, it will continue to adversely impact our business which could include, but not be limited to, negative impacts on income due to lower interest rates, lower lending, and transaction volumes, higher expected credit losses, lower wealth management revenue due to equity markets volatility and weakness, and increased model risk including credit loss models, capital models and asset/liability management models.
18. Our adjusted performance during 2020 decreased by $611 million compared with 2019 due primarily to a higher provision for credit losses driven by the deterioration of economic conditions caused by the COVID-19 pandemic and lower other revenues driven by lower trading revenue which were partially offset by lower operating expenses and higher net interest income.
19. Commercial loans decreased compared with December 31, 2019, due primarily to the impact of our efforts to improve returns through disciplined lending as well as lower demand from global banking clients reflecting increased capital markets activity coupled with economic uncertainty caused by the COVID-19 pandemic. Also contributing to the decrease were lower loans to affiliates. These decreases were partially offset by PPP loan originations in business and corporate banking. The decline in commercial non-affiliate loans was primarily in the diversified financial, real estate, energy, capital goods, and semiconductor industries.
20. Under workers’ compensation, we could experience a continuation of COVID-19 incurred losses, particularly due to laws or directives in certain states that require coverage of COVID-19 claims for health care and other essential workers based on a presumption that they contracted the virus while working. We could also incur losses on general liability policies if claimants can successfully assert that insureds were negligent in protecting employees, customers, and others from exposure.
